# The burden of non-alcoholic fatty liver disease among working-age people in the Western Pacific Region, 1990–2019: an age–period–cohort analysis of the Global Burden of Disease study

**DOI:** 10.1186/s12889-024-19047-y

**Published:** 2024-07-11

**Authors:** Jia-jie Lv, Yi-chi Zhang, Xin-yu Li, Hong Guo, Cheng-hao Yang

**Affiliations:** 1 Department of Vascular Surgery, School of Medicine, Shanghai Putuo People’s Hospital Tongji University, Huangpu District, No.1291 Jiangning Road, Shanghai, 200060 China; 2 Department of Vascular Surgery, Shanghai Ninth People’s Hospital, Shanghai Jiao Tong University, Shanghai, People’s Republic of China; 3 Department of Plastic and Reconstructive Surgery, Shanghai Ninth People’s Hospital Shanghai Jiao Tong University School of Medicine, No.639 Zhizaoju Road, Huangpu District, Shanghai, 200011 China; 4 Department of Gynecology and Obstetrics, Tongji Hospital, Tongji University School of Medicine, 389 Xincun Road, Putuo District, Shanghai, 200065 China

**Keywords:** Global burden of disease, Non-alcoholic fatty liver disease, Disability-adjusted life years, Age-period-cohort analysis, Trend analysis

## Abstract

**Background:**

The growing prevalence of non-alcoholic fatty liver disease (NAFLD) in younger populations, particularly those of working age (15–64 years), has become a public health concern. Being diagnosed at a younger age implies a greater likelihood of accruing disability-adjusted life years (DALYs) later in life due to potential progression to conditions such as cirrhosis or hepatocellular carcinoma. This study aims to analyze NAFLD prevalence trends over three decades globally, regionally, and nationally, with a focus on age, period, and birth cohort associations.

**Methods:**

Global, regional, and country time trends in the prevalence of NAFLD among working-age people from 1990 to 2019: Age-period-cohort analysis based on Global Burden of Disease Study 2019 estimates and 95% uncertainty interval (UI) of NAFLD prevalence in the working age population was extracted from the Global Burden of Diseases, Injuries and Risk Factors Study 2019. Age-period-cohort models were used to estimate the prevalence within each age group from 1990 to 2019 (local drift, % per year), fitted longitudinal age-specific rates adjusted for period bias (age effect), and period/cohort relative risk (period/cohort effect).

**Results:**

The global age-standardized prevalence (ASPR) of NAFLD increased significantly from 1990 (14,477.6 per 100 000) to 2019 (19,837.6 per 100 000). In the Western Pacific, there were 42,903.8 NAFLD cases in 2019, 54.15% higher than in 1990. The ASPR also increased significantly in the region over the past three decades. At the national level, Palau had the highest ASPR while Brunei Darussalam had the lowest. Age-period-cohort analysis showed that in the Western Pacific, unlike globally, the risk of NAFLD declined after age 60–64 years. Relative to 1980–1989, incidence and DALY risks decreased but prevalence increased in subsequent birth cohorts. Future predictions indicate an upward trend in NAFLD burden, especially among women and medium (SDI) regions like China.

**Conclusion:**

Non-alcoholic fatty liver disease imparts an immense health burden that continues to grow globally and in the Asia Pacific region. Our work highlights working age adults as an at-risk group and calls attention to socioeconomic gradients within Western Pacific countries. Upward future projections demonstrate that NAFLD prevention is an urgent priority.

**Supplementary Information:**

The online version contains supplementary material available at 10.1186/s12889-024-19047-y.

## Introduction

Nonalcoholic fatty liver disease (NAFLD) has a global prevalence of 25%, with a prevalence of approximately ≥ 34.0% in the US population and is a leading cause of cirrhosis and hepatocellular carcinoma [1, 2]. Among these cases of NAFLD, fewer than 10% develop cirrhosis or hepatocellular carcinoma 10 to 20 years after diagnosis. However, a significant proportion, including about one third of people with type 2 diabetes, have nonalcoholic steatohepatitis (NASH), which may progress more slowly or inappreciably over time [2, 3].Within the next decade, NAFLD is anticipated to become the predominant cause of liver cirrhosis necessitating transplantation. The spectrum of NAFLD ranges from simple steatosis to nonalcoholic steatohepatitis, liver fibrosis, and ultimately cirrhosis. The pathogenesis of NAFLD is intricate and influenced by multiple factors [4]. Recent initiatives propose redefining NAFLD, requiring the presence of at least one additional metabolic comorbidity for diagnosis [5, 6].

A recent meta-analysis has demonstrated a significant elevation in the prevalence of nonalcoholic fatty liver disease (NAFLD) in Asia, currently estimated at 29.6%, with substantial inter-country variability [7]. The Global Burden of Disease study has quantified the burden of NAFLD, employing the Regional and Sociodemographic Index (SDI) to assess data across 204 countries and territories from 1990 to 2019 [6]. Analysis of temporal trends in NAFLD during this period may facilitate the development of more effective, scientifically grounded preventive strategies. The most recent data from the Global Burden of Disease study indicates a prevalence of NAFLD of 69.9% among overweight individuals worldwide. Further scrutiny reveals that, while the prevalence of NAFLD in overweight populations of Asian and Middle Eastern countries stands at 59.7%, it remains markedly lower than that observed in European countries, and North and South America [8, 9].

Although previous studies have investigated the prevalence, incidence, and Disability-Adjusted Life Years (DALYs) associated with nonalcoholic fatty liver disease (NAFLD), there has been limited focus on changes within the working-age population (15–64 years). Utilizing data from the 2019 Global Burden of Disease study, we applied an age-period-cohort (APC) model to analyze trends in NAFLD prevalence among the global, regional, and national populations of working age from 1990 to 2019.

## Methods

### Data sources

The Global Burden of Disease (GBD) 2019 study represents a comprehensive international collaboration, supported by the Institute for Health Metrics and Evaluation and underpinned by sustained cross-country cooperation. This epidemiological endeavor has facilitated the estimation of the global burden for 369 diseases and injuries, alongside 87 risk factors, differentiated by age and sex across 204 countries and regions from 1990 to 2019 [10, 11]. For this analysis, we accessed and utilized repeated cross-sectional data, including numerical and age-standardized rates (ASRs), 95% uncertainty intervals (UI), and Disability-Adjusted Life Years (DALYs) for NAFLD incidence and prevalence, disaggregated by sex, age, region, and country over three decades in the Western Pacific region. Specifically, the age-standardized incidence rate (ASIR) indicates new cases per 100,000 population, the age-standardized prevalence rate (ASPR) denotes age-standardized cases per 100,000 population, and the age-standardized DALY rate (ASDR) represents age-standardized years lived with disability per 100,000 population. The 95% UI, derived from the 25th to the 75th ordered values of a 1,000-fold posterior distribution, reflects the range within which these estimates can be confidently made. Additionally, we gathered data on the Sociodemographic Index (SDI) for each country or region, calculated based on lagged per capita income distribution, average educational attainment for individuals aged 15 and older, and the Total Fertility Rate (TFR) for women under 25 years of age. This index, ranging from 0 to 1, serves as a proxy for the social and economic dimensions influencing health outcomes at each study site, with higher values indicative of superior socioeconomic conditions. Countries were categorized into five SDI quintile regions: low, medium–low, medium, high-medium, and high.

### Case Definition

According to the guidelines from the American Association for the Study of Liver Diseases (AASLD), non-alcoholic fatty liver disease (NAFLD) is characterized by hepatic steatosis, confirmed through imaging or histology, and excludes secondary causes of hepatic lipid accumulation such as significant alcohol intake, prolonged use of steatogenic medications, or other chronic liver diseases. Non-alcoholic fatty liver (NAFL) is described by the AASLD as the presence of hepatic steatosis without signs of hepatocellular injury, as evidenced by hepatocellular ballooning. NAFLD is diagnosed using imaging techniques such as MRI, CT, vibration-controlled transient elastography (VCTE), ultrasound, or definitively by biopsy. Consistent with the AASLD’s definition of NAFLD, studies that employed blood-based diagnostic markers for NAFLD, which have lesser accuracy, were not included in our analysis. Overweight is defined as a body mass index (BMI) of 25.0 kg/m^2^ or greater for the general population and 23.0 kg/m^2^ or greater for Asian individuals; obesity is defined as a BMI of 30.0 kg/m^2^ or greater for the general population and 27.5 kg/m^2^ or greater for Asian individuals. The prevalence of NAFL and non-alcoholic steatohepatitis (NASH) is conclusively determined only through liver biopsy. Staging of liver fibrosis is conducted by assessing histopathological features (stages F1–F4) from biopsies, with clinically significant fibrosis quantified as stages F2 to F4 and advanced fibrosis as stages F3 to F4 [12, 13].

### Sociodemographic Index

The Sociodemographic Index (SDI) is a composite indicator reflecting a country’s or region’s level of developmental progress [14]. The SDI is constructed using the geometric mean of three components: per capita income, mean educational attainment among individuals aged 15 years and older, and the total fertility rate. In the GBD 2019, countries and territories were stratified into five SDI categories: low (< 0.46), low-middle (0.46–0.60), middle (0.61–0.69), high-middle (0.70– 0.81), and high (> 0.81). It is important to note that lower SDI values are indicative of lesser degrees of societal development [15].

### Statistical analysis

#### Overall time trend analysis of NAFLD prevalence in working age population

The first aim of this study was to investigate the temporal trends in the incidence, prevalence, DALYs and mortality of NAFLD from 1990 to 2019. To quantify these effects, we used a set of indicators including incidence, prevalence, DALY, and mortality, each supplemented by a corresponding rate. Disability-adjusted life years (DALYs) are a composite measure of the burden of disease that includes years of life lost due to premature death and years of life lost due to disability. Burden was carefully estimated and articulated and set within 95% uncertainty intervals (UIs) to maximize precision. For a detailed understanding of the methodology employed, we refer the reader to the relevant academic literature [16–18]. Given the different age distributions and demographic characteristics in the Global Burden of Disease (GBD) dataset, it is critical to adjust for different age structures. To do so, we calculated age-standardized incidence rates (ASR) per 100,000 using the following formula[19]:

$$\mathrm{ASR}=\frac{\sum_{\mathrm i=1}^{\mathrm A}{\mathrm a}_{\mathrm i}{\mathrm w}_{\mathrm i}}{\sum_{\mathrm i=1}^{\mathrm A}{\mathrm w}_{\mathrm i}}\times10,000$$

(a_i_: the age-specific rate in ith the age group; w: the number of people in the corresponding i.th age group among the standard population; A: the number of age groups) To examine the temporal patterns of incidence, mortality, and DALYs, we calculated the Estimated annual percentage change (EAPC) rates. The EAPC serves as a prevalent metric in epidemiological studies to ascertain temporal evolutions in ASRs of diseases. The coefficient, denoted as β, is derived from the natural logarithm of the ASRs. Herein, y represents ln(ASR) while. x corresponds to the calendar years. The EAPC, accompanied by its 95% confidence interval (CI), was determined utilizing the ensuing linear regression model:

$$\mathrm y=\mathrm\alpha+\beta x+\mathrm\varepsilon$$

$$\mathrm{EAPC}=100\ast(\exp\left(\beta\right)-1.$$

The trend of the ASR can be discerned by analyzing the EAPC and its corresponding 95% CI. If the EAPC value and the lower limit of the 95% CI are both positive, this indicates an upward trend in the ASR. Conversely, if both the EAPC value and the upper limit of the 95% CI are negative, this suggests a downward trend in the ASR [14]. To predict the future disease burden from 1990 to 2045, we utilized a log-linear age-period-cohort model. This model restricts linear trend projection and curbs exponential growth, rendering it suitable for fitting recent trends. We implemented the model in R using the NORDPRED package. To explore the factors influencing the changes of disease burden, the relationships between ASRs and SDI were calculated globally and in 20 geographic regions using Pearson’s correlation analysis from 1990 to 2019.

#### Age–period–cohort analysis

The second objective was to conduct an age-period-cohort (APC) analysis to assess the different effects of age, period, and birth cohort on NAFLD incidence, prevalence, and DALY rate. The age factor reflects the social and biological dynamics of aging. Period effects refer to the effects of events and changes, such as updates in diagnostic criteria or advances in treatment, on the statistics for bipolar disorder across age groups. Cohort effect refers to the changes of disease impact caused by different exposure levels of risk factors in different population generations. The age-period-cohort analysis in our study was conducted using the APC Web Tool14, which is developed by the Biostatistics Branch of the National Cancer Institute in Bethesda, Maryland. This tool is accessible online at [ http://analysistools.nci.nih.gov/apc/] [20] . Key parameters include:(a) net drift, representing the overall annual percentage change by calendar year and birth cohort in a log-linear fashion; (b) Local drift, with log-linear trends for each age group expressed by calendar year and birth cohort, detailing the annual percentage change for each age group; (c) longitudinal age curves showing adjusted longitudinal age-standardized rates (ASR) in the reference cohort, accounting for period bias; (d) Period relative risk (RR), time-related risk relative to the reference period, adjusted for age and nonlinear cohort effects; (e) Cohort RR, comparing the risk of the birth cohort relative to the reference cohort, adjusting for age and nonlinear period effects [21]. Wald chi-square tests were used to determine the significance of estimable parameters and functions. We report the overall age, period, and cohort effects of NAFLD in the Western Pacific region and further disaggregate these effects by sex. Analyses and graphical representations were performed with the use of R statistical software, version 4.3.0. Two-sided p values of less than 0.05 were considered to indicate statistical significance.

## Results

### Trends in NAFLD in working-age individuals, 1990–2019

#### Global trends

In 1990, there were 77,749 cases of nonalcoholic fatty liver disease (NAFLD) among working-age individuals (95% uncertainty interval [UI], 31,773.5 to 153,582.4). By 2019, this number had escalated to 145,204.6 cases (95% UI, 61,383.6 to 282,231.7), representing an 86.76% increase over the period. Notably, the age-standardized incidence rate (ASIR) demonstrated a significant rise from 1990 to 2019, with an estimated annual percentage change (EAPC) of 0.55 (95% confidence interval [CI], 0.41 to 0.69) as shown in Table 1. Additionally, the estimated global prevalence of NAFLD in 2019 was approximately 1,002,886,383 cases (95% UI, 742,743,853 to 1,298,691,912), an increase of 111.79% since 1990, with the global age-standardized prevalence rate (ASPR) also significantly rising from 14,477.6 per 100,000 in 1990 to 19,837.6 per 100,000 in 2019 (EAPC = 1.1; 95% CI, 1.0 to 1.2) as detailed in Table S1. Furthermore, the estimated global disability-adjusted life years (DALYs) attributable to NAFLD in 2019 were approximately 2,913,515 (95% UI, 1,677,499 to 4,646,107), marking a 49.36% increase since 1990, as documented in Table S2. In terms of mortality, the number of deaths in people with NAFLD increased from 49,236.1 in 1990 to 75,325 in 2019, but the age-standardized mortality rate decreased (EAPC = -0.23, 95%CI -0.4–0.06, Table S3). The number of nonalcoholic fatty liver disease (NAFLD) cases was highest among individuals aged 45 to 49 years, as depicted in Figures S1A and S1B. Conversely, the highest rates of disability-adjusted life years (DALYs) and mortality were observed in those aged 60 to 64 years, as shown in Figures S1C and S1D. Additionally, the incidence and prevalence of NAFLD escalated across all age groups, peaking among individuals aged 55 to 59 years and 60 to 64 years, respectively, according to Figures S2A and S2B. The most substantial DALY rates were noted in the age group of 60 to 64 years, followed by those aged 55 to 59 years, with a similar pattern evident in mortality rates, detailed in Figures S2C and S2D.

**Table 1:** Incidence of NAFLD from 1990 to 2019 in individuals aged 15 to 64 years globally and in the Western Pacific Region

Location	1990		2019		EAPC_95%CI
	Number_95%UI	ASIR	Number_95%UI	ASIR	
Global	77,749 (31,773.5–153,582.4)	2.4 (1–4.7)	145,204.6 (61,383.6–282,231.7)	2.9 (1.2–5.6)	0.55 (0.41–0.69)
Western Pacific Region	27,833.4 (12,094.9–53,974.9)	2.7 (1.2–5.2)	42,903.8 (18,498.8–82,973.9)	3.2 (1.4–6.1)	0.38 (0.1–0.67)
Australia	238.4 (96.8–461.8)	2.1 (0.9–4.1)	408.6 (177.9–771.5)	2.6 (1.1–4.8)	0.83 (0.67–1)
Brunei Darussalam	1.7 (0.7–3.3)	1.1 (0.4–2)	4.2 (1.9–7.9)	1.3 (0.6–2.4)	0.14 (-0.07–0.35)
Cambodia	228.4 (63.9–515)	4.3 (1.2–9.7)	649.4 (204.5–1415.9)	6.1 (1.9–13.3)	0.99 (0.72–1.26)
China	23,046.5 (9686.3–45,310.9)	2.9 (1.2–5.7)	34,551.2 (14,523.8–67,625)	3.4 (1.4–6.6)	0.34 (-0.01–0.69)
Cook Islands	0.1 (0.1–0.2)	1.2 (0.6–2.1)	0.2 (0.1–0.4)	1.8 (0.9–3.2)	1.56 (1.47–1.65)
Fiji	5.4 (2–10.8)	1.2 (0.5–2.4)	9.4 (4–18.1)	1.6 (0.7–3.1)	1.07 (1.02–1.12)
Japan	1666.3 (819.8–3030.9)	1.9 (0.9–3.5)	1045.7 (498.6–1923)	1.4 (0.7–2.5)	-1.23 (-1.38–1.08)
Kiribati	1 (0.3–2)	2.3 (0.8–4.8)	1.9 (0.7–3.8)	2.6 (1–5.2)	0.19 (0.12–0.26)
Lao People's Democratic Republic	45.5 (15.4–96.8)	2.1 (0.7–4.5)	121 (43.8–252.6)	2.6 (1–5.5)	0.55 (0.28–0.82)
Malaysia	147.9 (59.2–289.5)	1.4 (0.6–2.8)	497.6 (216–947.3)	2.3 (1–4.4)	1.51 (1.33–1.69)
Marshall Islands	0.4 (0.1–0.9)	1.8 (0.6–3.9)	0.9 (0.3–1.7)	2.4 (0.9–4.8)	0.76 (0.6–0.92)
Micronesia (Federated States of)	1.1 (0.4–2.3)	2 (0.7–4.3)	1.7 (0.7–3.5)	2.6 (1–5.2)	0.76 (0.69–0.84)
Mongolia	53.4 (22.8–101.5)	4.6 (1.9–8.7)	257.4 (122.1–468)	11.5 (5.5–20.9)	3.8 (3.55–4.04)
Nauru	0.1 (0–0.2)	1.8 (0.7–3.8)	0.2 (0.1–0.3)	2.3 (0.9–4.7)	0.68 (0.54–0.82)
New Zealand	40.6 (13.7–84.4)	1.8 (0.6–3.8)	70.2 (30.5–131.2)	2.5 (1.1–4.6)	0.99 (0.91–1.07)
Niue	0 (0–0)	1.7 (0.7–3.4)	0 (0–0.1)	2.5 (1–4.8)	1.15 (0.87–1.42)
Palau	0.2 (0.1–0.3)	1.7 (0.7–3.5)	0.4 (0.2–0.7)	2.9 (1.2–5.6)	1.64 (1.32–1.96)
Papua New Guinea	10.8 (3.1–24.8)	0.5 (0.1–1.1)	33.6 (10.2–74.9)	0.6 (0.2–1.3)	0.61 (0.57–0.65)
Philippines	520.6 (185.1–1104.1)	1.5 (0.5–3.1)	1654.6 (582–3453.9)	2.3 (0.8–4.9)	1.78 (1.62–1.94)
Republic of Korea	519 (263.7–901.4)	1.7 (0.9–2.9)	724.7 (366.7–1263.1)	1.9 (0.9–3.3)	-0.03 (-0.59–0.53)
Samoa	1.5 (0.5–3)	1.6 (0.6–3.4)	2.4 (0.9–5)	1.9 (0.7–3.9)	0.54 (0.31–0.77)
Singapore	21.6 (9.3–41.1)	1 (0.4–1.8)	39.9 (17.9–75.1)	0.9 (0.4–1.8)	-0.62 (-0.82–0.42)
Solomon Islands	3.7 (1.2–8)	2.1 (0.7–4.6)	9.5 (3.2–20.2)	2.5 (0.9–5.3)	0.55 (0.36–0.75)
Tonga	1.5 (0.6–2.8)	2.8 (1.2–5.3)	2.3 (1.1–4.3)	3.9 (1.8–7.3)	0.98 (0.72–1.24)
Tuvalu	0.1 (0–0.2)	1.6 (0.6–3.3)	0.1 (0.1–0.3)	1.9 (0.8–3.8)	0.41 (0.35–0.48)
Vanuatu	1.9 (0.6–4)	2.3 (0.7–5.1)	4.5 (1.6–9.3)	2.7 (0.9–5.4)	0.46 (0.37–0.56)
Viet Nam	766.2 (266.5–1634.4)	2 (0.7–4.3)	2217 (828–4490.4)	3.2 (1.2–6.6)	1.59 (1.18–2.01)

*Abbreviations*: *NAFLD* Non-alcoholic fatty liver disease, *ASIR* Age-Standardized Incidence Rate, *EAPC* Estimated annual percentage change, *UI* Uncertainty interval

#### Regional trend

In the Western Pacific region, the number of nonalcoholic fatty liver disease (NAFLD) cases in 2019 was 42,903.8 (95% uncertainty interval [UI], 18,498.8–82,973.9), representing a 54.15% increase from 1990 (27,833.4 cases; 95% UI, 12,094.9–53,974.9). Although the age-standardized incidence rate (ASIR) of NAFLD rose over the three decades, the increase was less pronounced than the global trend, with an estimated annual percentage change (EAPC) of 0.38 (95% confidence interval [CI], 0.1–0.67), as shown in Table 1. Furthermore, the prevalence of NAFLD in the Western Pacific region experienced a significant surge over the same period. In 1990, the prevalence was recorded at 141,926,763.8 cases (95% UI, 100,909,536.3–190,924,133.4), escalating to 283,234,498 cases by 2019 (95% UI, 208,499,833.1–367,325,048.3), an increase of 99.56%, as detailed in Table S1. The age-standardized prevalence rate (ASPR) also saw a substantial rise over the past three decades, with an EAPC of 1.48 (95% UI, 1.21–1.74). Concurrently, there was a reduction in both the disability-adjusted life year (DALY) rate and the number of NAFLD-related deaths, decreasing by 7.41% and 4.04% respectively, over the 30-year period, as indicated in Tables S2 and S3.

Gender-based analyses reveal that both the age-standardized incidence rate (ASIR) and the age-standardized prevalence rate (ASPR) of nonalcoholic fatty liver disease (NAFLD) were higher in men than in women in the Western Pacific region. However, the male-to-female ratio declined with age, reaching its peak in the 30–34 year age group. Notably, the ASIR for women exceeded that of men starting in the 40–44 year age group. Despite these shifts in incidence, the ASPR of NAFLD consistently remained higher in men across all age groups, as depicted in Figures S3A and S3B. Additionally, the trends in age-standardized disability-adjusted life year (DALY) rates and age-standardized mortality rates showed similar patterns, peaking in the 35–39 year age group, with the lowest rates observed in the 150–190,000 age group. Moreover, in all age groups, both the age-standardized DALY rates and age-standardized mortality rates were consistently higher in males than in females, as illustrated in Figures S3C and S3D.

#### SDI trends

From 1990 to 2019, the estimated number of cases within the Sociodemographic Index (SDI) categories increased across all five regions of the Western Pacific Region. For this study, five countries were selected to represent varying SDI categories: Australia for the high SDI, China for the high-medium SDI, Fiji for the medium SDI, Nauru for the medium–low SDI, and Cambodia for the low SDI. Figures S3 and S4 illustrate the male-to-female ratios for each measure of nonalcoholic fatty liver disease (NAFLD) within each SDI region. For the age-standardized incidence rate (ASIR), in high SDI regions, the male-to-female ratio gradually increased with age, stabilizing after the age of 45. The male-to-female ratio surpassed that of females for the first time in the 45–49 age group and peaked in the 50–54 age group. In contrast, in the remaining four SDI regions, the male-to-female ratios declined. Notably, the male-to-female ratio peaked in the 30–34 age group in the medium–high, medium, and medium–low SDI regions. In the high-medium, medium, and low SDI areas, the proportion of women exceeded that of men starting in the 45–49 age group, while in the medium–low SDI area, this shift occurred after the 50–54 age group. For the age-standardized prevalence rate (ASPR), all regions except the high SDI showed a declining trend, particularly notable in the 15–29 age group, stabilizing after age 29. In high SDI areas, the gender ratio remained relatively unchanged until age 44, with the ASPR of males being more than twice that of females. After age 44, although the gender ratio decreased significantly, the ASPR of males continued to exceed that of females. Additionally, the age-standardized DALY rates and mortality rates displayed similar trends in men and women across each SDI, characterized by an inverted U-shape (Figure S3). Over these 30 years, the incidence, prevalence, and DALY rates of NAFLD increased across all SDI regions (Figure S5). Notably, the ASIR and DALY rates in low SDI regions were significantly higher than the average in the Western Pacific region and have rapidly increased over the past decade. Intriguingly, the ASPR in the medium SDI region, represented by Fiji, was the highest among the five regions and continued to rise (Figure S5).

#### National trends

This study analyzed the prevalence trends of NAFLD in working-age adults (15–64 years) in the Western Pacific region from 1990 to 2019 and found significant differences between countries. In 2019, Mongolia reported the highest age-standardized incidence rate of NAFLD among Western Pacific countries, at 11.5 cases per 100,000 people (95% uncertainty interval [UI]: 5.5 to 20.9, Table 1). In contrast, Papua New Guinea had the lowest incidence of 0.6 cases per 100,000 population (95% UI, 0.2 to 1.3; Table 1). During the same period, the greatest increase in NAFLD incidence was observed in the Philippines (EAPC:1.78; 95% confidence interval [CI]: 1.62 to 1.94), with the largest decrease observed in Japan (EAPC: -1.23; 95%CI: -1.38 – 1.08). For ASPR, in 2019, the prevalence of NAFLD in Palau was the highest among the Western Pacific countries surveyed, at 30,251.3 cases per 100 000 people (95%UI: 22,729.5 to 38,505.9, Table S1). In sharp contrast, Brunei Darussalam reported the lowest prevalence, actually 10,578.7 cases per 100 000 (95% UI: 7565.7 to 14,052, Table S1). From 1990 to 2019, the Republic of Korea had the greatest increase in NAFLD prevalence (EPAC:1.61; 95% CI: 1.22 to 2.01, Table S1), whereas Nauru had the smallest reduction (EAPC: 0.22; 95% CI: 0.14–0.29, Table S1).

**Fig. 1:**
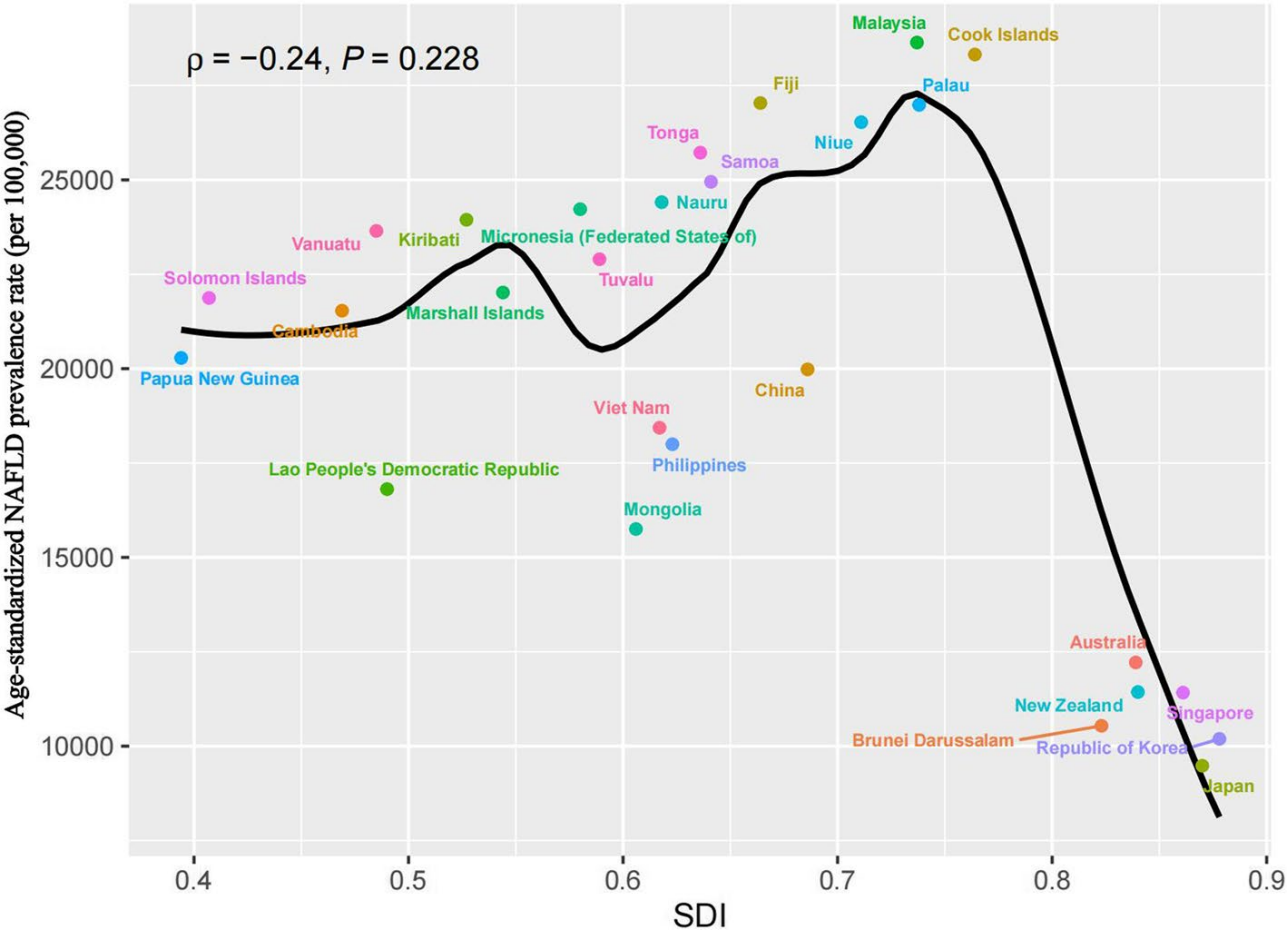
Association between Age-standardized NAFLD Prevalence Rate and Sociodemographic Index

In addition, Mongolia, Cambodia, and Kiribati had the highest age-standardized DALY of NAFLD among Western Pacific countries in 2019, with 240.9, 179.9, and 122.8 cases per 100 000 population, respectively (Table S2). In contrast, Singapore, Japan and Brunei Darussalam reported the lowest ASDR at 9, 14.7 and 22.3 per 100 000 population, respectively (Table S2). From 1990 to 2019, Mongolia, Palau and Australia had the most significant increases in age-standardized DALY rate (EPAC: 2.01, 1.87 and 1.52, respectively). In contrast, Japan, China, and the Lao People's Democratic Republic experienced the largest declines (EPAC: -2.9, -2.55, and -1.31, respectively; Table S2). For age-standardized mortality rates, Mongolia reported the highest age-standardized mortality rate for NAFLD among Western Pacific countries, at 6.5 cases per 100 000 population (95% uncertainty interval [UI]: 3.5–10.7, Table S3). In contrast, Singapore had the lowest mortality rate, at 0.3 cases per 100,000 (95% UI, 0.2 to 0.4; Table S3). Figure 1 and S6–8 shows that ASIR, ASDR and age-standardized mortality were significantly negatively correlated with SDI in each country in the Western Taiping region (P < 0.05). In addition, ASPR was also negatively correlated with SDI, but P > 0.05.

#### Age, period and cohort effects on the regional trend

**Fig. 2:**
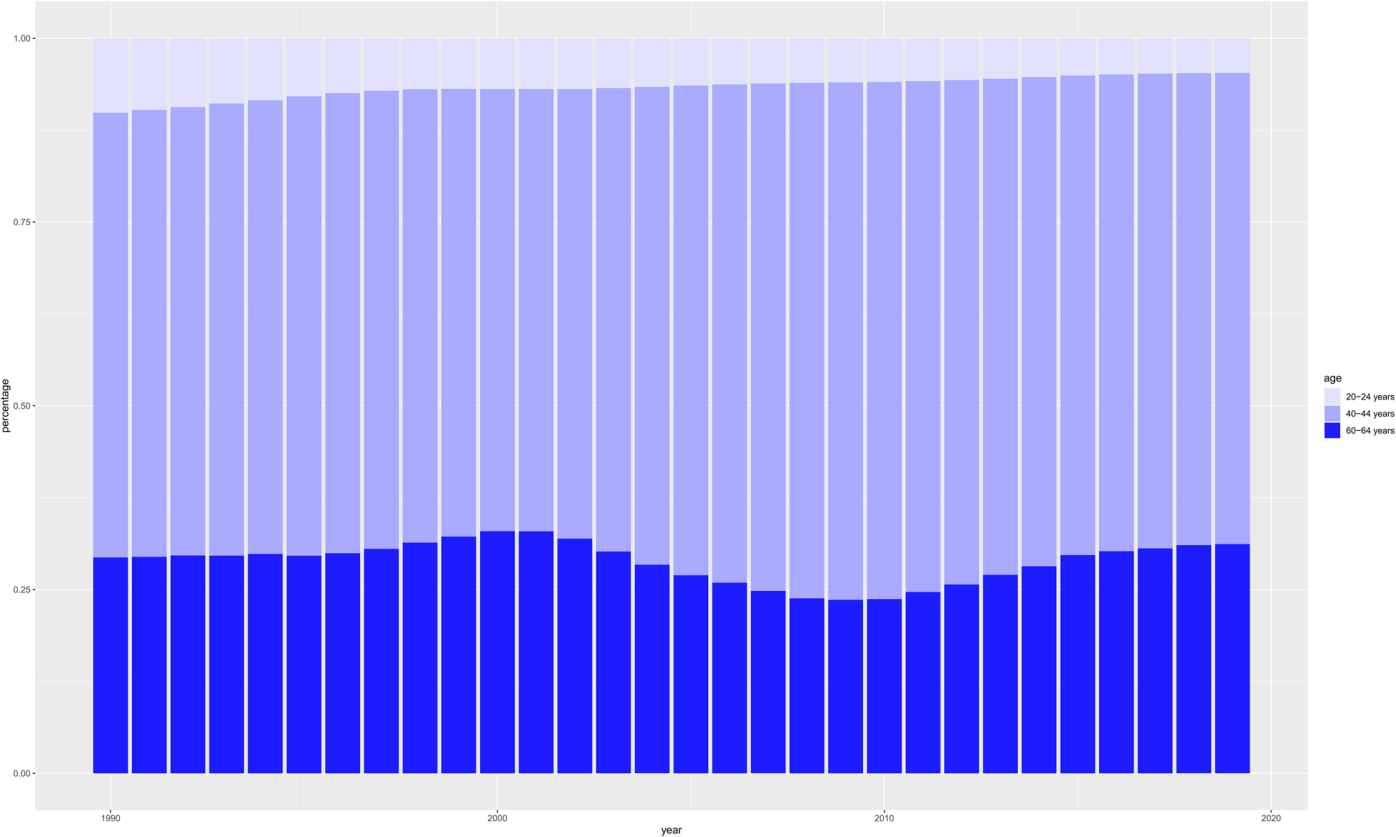
Temporal changes in the age distribution of NAFLD prevalence among working-age individuals from 1990 to 2019

**Fig. 3:**
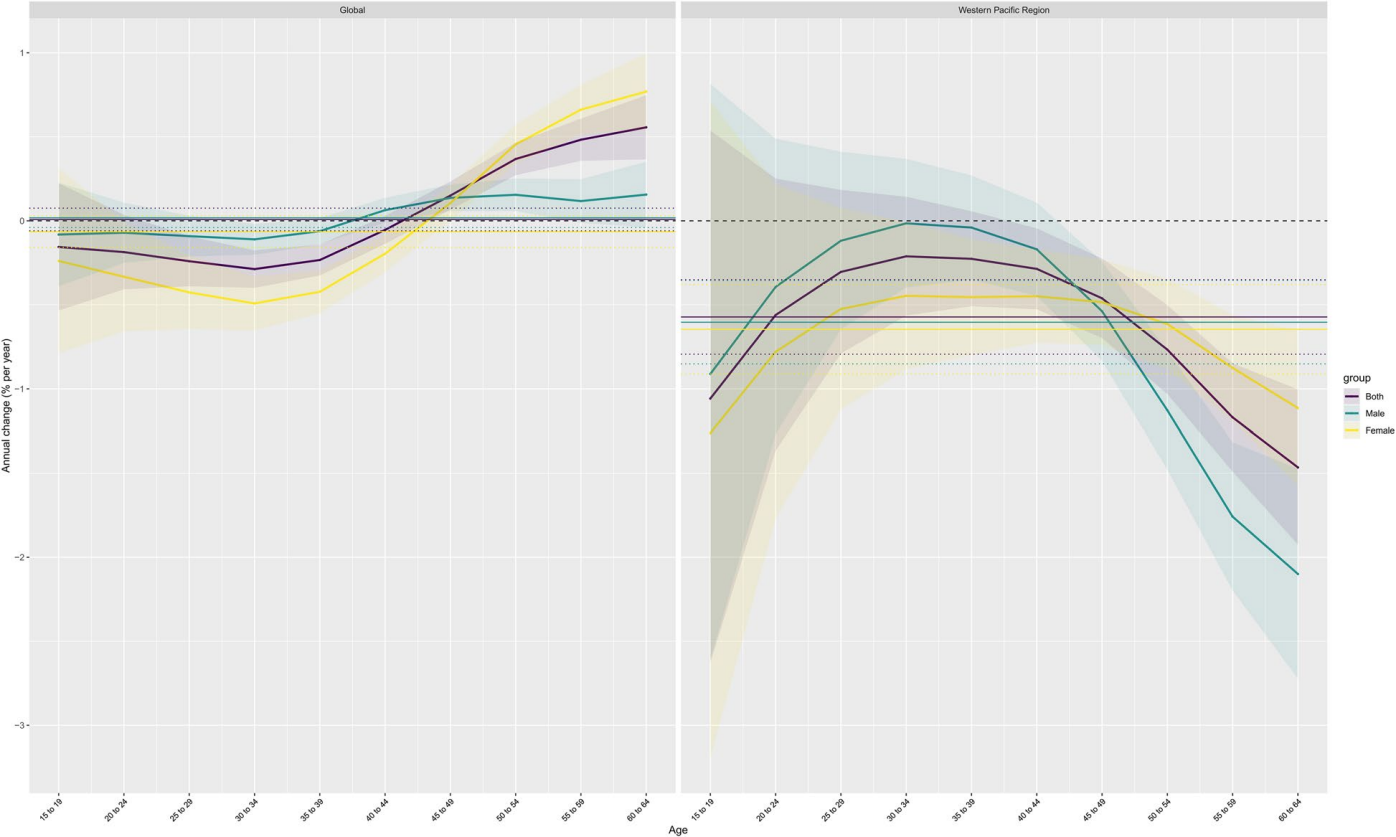
Local drift in NAFLD incidence globally and in the Western Pacific region from 1990 to 2019

**Fig. 4:**
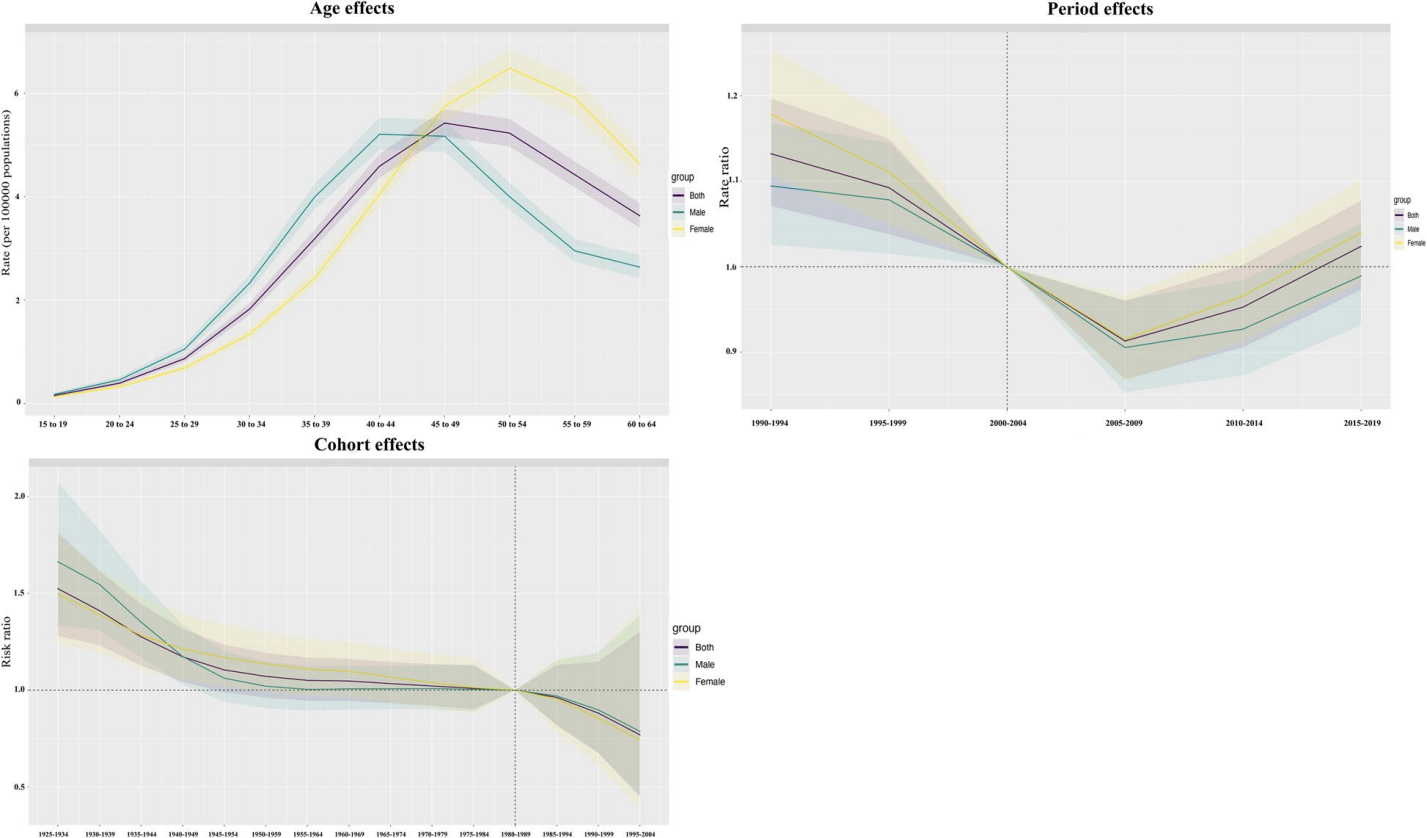
Age effect, period effect, and cohort effect on NAFLD incidence in the Western Pacific from 1990 to 2019

From 1990 to 2019, among working-age people, the risk of NAFLD in the Western Pacific Region first increased and then decreased with age, a trend that, interestingly, was contrary to the global trend (Figs. 2, 3 and Figure S9-10). In the Western Pacific, the decline was steeper among men (− 2.1 for those aged 60 to 64; 95% CI -2.72 to -1.47) (Table S4). Similar trends were observed among women (at age 60 to 64 years, − 1.11; 95% CI -1.57, -0.66)(Table S4, Fig. 3, S11-12). The prevalence and risk of NAFLD increased first and then decreased with age regardless of sex (Fig. 3, S11-12; Table S4-7). The risk of bipolar disorder was highest at 30 to 34 years of age for both women and men (Fig. 4A; Table S5). Among both women and men, the effect of age on prevalence was most pronounced between the ages of 25 and 29 years (Fig. 4, S13-14; Tables S6 and S7). The DALY rate generally decreased with age in both men and women, but increased after the age group of 40–44 years (Fig. 4, S13-14; Tables S8 and S9). The greatest age effects on the DALY rate were observed among men 20 to 24 years of age and women 15 to 19 years of age (Fig. 4, S13-14; Tables S8 and S9).

The period effect showed that the risk of incidence rate and DALY rate decreased with the increase of years in both men and women from 1990 to 2019, but the risk of disease showed an increasing trend (Table S10-12, Fig. 4, S13-14). Relative to the period from 2000 to 2004, the prevalence peaked between 2015 and 2019, and the incidence was highest between 1990 and 1994 (Table S10, S11). DALY risk peaked between 1990 and 1994 (Table S12, Fig. 4, S13-14).

From 1925–1934 to 1995–2004, cohort risks in successive 10-year birth cohorts fluctuated slightly overall. Compared with the 1980–1989 cohort, incidence rates and DALY rates in the cohort after 1985 decreased, but prevalence increased (Fig. 4, S13-14, P < 0.05). Table S13-S15). The incidence rate and DALY risk in the earlier birth cohort (before 1980) were higher than those in the reference birth cohort (1980–1989). In the cohort before the 1980 to 1989 reference group, men had lower risks for all three measures than women. However, in the most recent cohorts, the risk among men equals or exceeds that among women (Fig. 4, S13-14, Tables S13-S15).

**Fig. 5:**
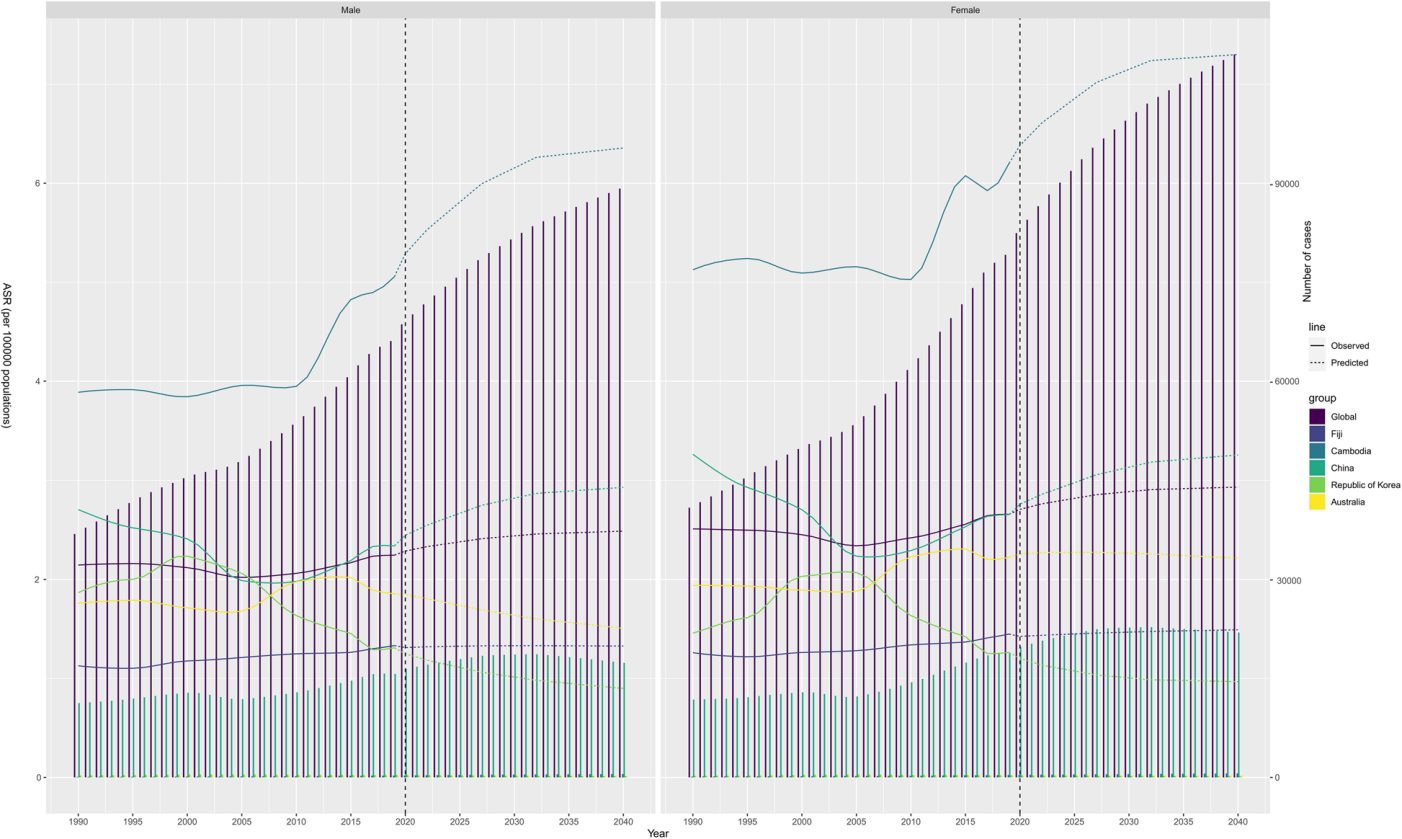
Future Forecasts of NAFLD Prevalence from the Global Burden of Disease Study

Rising Burden of NAFLD in the Western Pacific Region Fig. 5 plots the predicted trajectory of NAFLD in the working age population of the Western Pacific region, indicating an overall upward trend in the global burden of these diseases. It is worth noting that the prevalence of low SDI regions represented by Cambodia was significantly higher than the global prevalence and the growth rate was significant. In contrast, growth in other regions and countries has been more stable. In addition, gender differences were evident, with more women affected than men (Fig. 5). The predicted increase in NAFLD prevalence by 2040 will urgently require global health efforts and policy development.

## Discussion

This study offers a detailed examination of the burden of non-alcoholic fatty liver disease (NAFLD) among the working-age population globally, with a particular emphasis on countries within the Western Pacific Region from 1990 to 2019. Utilizing data from the Global Burden of Disease Study 2019, we analyzed trends in incidence, prevalence, disability-adjusted life years (DALYs), and mortality. An age-period-cohort analysis was conducted to delineate the effects of age, time period, and birth cohort on these trends. Our findings reveal a significant and increasing burden of NAFLD, identify populations at risk, and underscore the importance of targeted prevention and control strategies.

### Key findings

Our study found that the global burden of non-alcoholic fatty liver disease (NAFLD) has increased significantly over the past three decades. The number of new cases among working-age individuals nearly doubled from 1990 to 2019. Similarly, the estimated global prevalence in 2019 was more than double that of 1990, showing an increase of over 111%. Although there were increases in disability-adjusted life years (DALYs) and the number of deaths, the age-standardized mortality rate exhibited a slight decline. These findings are consistent with other studies that report a rising global prevalence of NAFLD, underscoring the urgent need for population-level interventions [22]. Specifically, in the Western Pacific Region, our analysis reveals more than a 54% growth in incident cases between 1990 and 2019, with prevalence estimates nearly doubling during the same period. However, unlike the global trends, we observed a decrease in DALY rates and mortality in this region. This disparity suggests regional variations in risk factors, diagnostic accuracy, disease progression, or management of NAFLD that require further exploration. The escalation in NAFLD burden is associated with changes in risk factors such as population aging, urbanization, obesity, and diabetes. Previous research has linked abdominal obesity and components of the metabolic syndrome to an increased risk of NAFLD [23]. Economic improvements and lifestyle shifts in many Western Pacific countries may be contributing to the rise in metabolic disorders and NAFLD. Effective public health measures are essential to curb the further increase projected by our study.

### Age Patterns

Our age-period cohort analysis provided additional insights into the evolving burden of NAFLD. Globally, NAFLD risk increased with age over the life course. However, in the Western Pacific Region, we observed an inverted U-shaped trend, with NAFLD incidence, prevalence, and disability-adjusted life years (DALYs) declining after middle age. This pattern indicates a relatively early onset of the disease, followed by a plateau or decline, suggesting earlier peak prevalence between ages 50 to 59, as documented in prior studies of the Asia Pacific region [24]. A possible explanation for this decline could be survivor bias, where NAFLD-related mortality increases at older ages. Additionally, cohort effects may play a role if younger generations are experiencing greater cumulative exposure to risk factors for NAFLD. Independent of geographic location, the risk and burden of NAFLD begin to rise in young adulthood, highlighting the importance of early screening and preventive measures. Specifically, in the Western Pacific, interventions targeted at individuals under 60 years of age could significantly reduce the population burden of NAFLD. Lifestyle interventions, including diet and exercise, should commence early and persist through middle age, where susceptibility to NAFLD appears to be highest.

### Period and cohort effects

From 1990–2019, our period analysis indicates decreasing incidence rate and DALY rate over time in the Western Pacific. However, NAFLD prevalence increased. This growing burden, despite a declining incidence, suggests that individuals may be living longer with the disease, likely due to advancements in management. Improved screening and diagnostics over time, as well as potentially increased accuracy of health metrics in recent years, may also contribute to these trends [4, 25]. Analysis of successive birth cohorts reveals fluctuations in NAFLD risk, characterized by general declines in incidence and DALYs, but increases in prevalence among recent cohorts. A lower incidence may reflect reduced exposure to risk factors among younger generations or the adoption of healthier lifestyles. Concurrently, enhanced awareness and detection of existing cases may elevate prevalence figures. These complex dynamics across age, period and birth cohorts likely involve interactions between genetic risks, epigenetic changes, and variable environmental triggers [26].

### Sex differences

In examining NAFLD measures stratified by sex, we found higher age-standardized incidence and prevalence among males compared to females in the Western Pacific. However, the male-to-female ratio decreased with age. After age 40, incidence was actually higher in women. This aligning of incidence likely relates to hormonal changes during menopause that shift abdominal fat distribution and metabolic profiles. Accordingly, studies project a narrowing sex difference in NAFLD prevalence at older ages as postmenopausal women catch up to men [27]. Sex hormones like estrogen may be protective earlier in life, partially explaining lower female NAFLD risk [28]. However, women face additional susceptibility from gestational exposures and multiparity [29]. Premenopausal, overweight women with a history of gestational diabetes have markedly higher odds of NAFLD [30]. Overall, our findings highlight females as an at-risk group warranting greater clinical attention and research. Preventive health services should target reproductive-aged women to curb incident NAFLD.

### Socioeconomic inequalities

This analysis also exposes socioeconomic gradients in NAFLD within Western Pacific countries. Age-standardized incidence, DALYs and mortality declined significantly as socioeconomic development increased based on Sociodemographic Index (SDI) quintiles. Prevalence showed a similar inverse trend. Cambodia, representing low SDI, faced the highest mortality whereas Singapore had the lowest. Poorer nations likely experience higher NAFLD due to constrained health systems, limited diagnostics, and inadequate prevention resources [31]. Conversely, economic improvement may foster lifestyle changes that exacerbate metabolic diseases. Notably, Fiji, a nation with a middle-tier SDI, demonstrated a unique and escalating prevalence of NAFLD during the study period. Pacific Island communities are experiencing rapid epidemiological transitions characterized by increasing obesity and diabetes rates, reflecting shifts towards Western dietary and activity patterns conducive to NAFLD. However, health services in these regions remain inadequate. Addressing this disproportionate burden will necessitate substantial investments in chronic disease prevention and management, specifically tailored to the cultural contexts of these communities.

### Future projections

Our modeling predicts steady increases in the future prevalence of NAFLD globally and in the Western Pacific Region if current trends continue. However, countries in the low to middle SDI range face particularly steep trajectories. By 2040, NAFLD prevalence in regions like Mongolia and China is expected to far surpass global estimates and peers. Realizing these projections without intervention could overwhelm health systems. There is a narrowing window to implement policy and infrastructural changes that promote healthy lifestyles on a population scale. Success necessitates cooperation across government sectors and engagement with the international community [32, 33].

### Limitations

Findings should be interpreted in light of data limitations. Recent proposals suggest redefining NAFLD to mandate the presence of at least one other metabolic comorbidity for diagnosis. In light of this context, the analysis would benefit from incorporating anthropometric and laboratory data on measures of adiposity, glucose metabolism, blood pressure, and dyslipidemia in the studied cohort [5]. Specifically, body mass index, waist circumference, fasting glucose and insulin, hemoglobin A1c, serum lipids, and resting blood pressure are relevant variables that provide insight into metabolic health. Additionally, directly analyzing the relationship between metabolic traits and NAFLD presence and severity may reveal meaningful patterns that further current mechanistic understanding [5, 6]. The inputs relied on observational studies and modeled estimates. The accuracy of Global Burden of Disease metrics depends on the completeness of country reporting systems. Under-diagnosis likely contributes to underestimates, particularly in nations with minimal screening. Variable access to liver biopsies, imaging, and elastography for definitive NAFLD diagnosis can also distort disease estimates [4]. At the same time, differences in case definitions over time and between locations reduce comparability. For example, overweight body mass index (BMI) thresholds for Asian populations further complicate global comparisons. These constraints emphasize the need for capacity building and standardized surveillance.

## Conclusions

Non-alcoholic fatty liver disease (NAFLD) represents a significant and escalating health burden globally and particularly in the Asia Pacific region. Our research identifies working-age adults as a demographic particularly vulnerable to this condition and underscores the socioeconomic disparities observed within countries of the Western Pacific. Projections indicate that the prevention of NAFLD must be regarded as an urgent global health priority. Immediate, coordinated action across multiple sectors is crucial to mitigate the increasing prevalence of this disease. Such actions should encompass the implementation of policies aimed at reducing obesogenic environments, enhancing community education, establishing rigorous screening protocols, and upgrading healthcare infrastructure, especially in settings with limited resources. Additionally, adopting a life course approach to interventions could substantially alter the trajectory of NAFLD for both present and future generations.

### Supplementary Information

Supplementary Material 1.

## Data Availability

No datasets were generated or analysed during the current study.

## Abbreviations

NAFLD: Non-alcoholic fatty liver disease
DALYs: Disability-adjusted life years
GBD: Global burden of disease
SDI: Socio-demographic index
EAPC: Estimated annual percentage changes
ASR: Age-standardized rate
ASIR: Age-standardized incidence rate
ASPR: Age-standardized prevalence rate
ASDR: Age-standardized DALY rate
CI: Confidence interval
UI: Uncertainty intervals
