# Supplementary material for: The burden of non-alcoholic fatty liver disease among working-age people in the Western Pacific Region, 1990–2019: an age–period–cohort analysis of the Global Burden of Disease study
Source: BMC Public Health. 2024 Jul 11;24:1852. doi: 10.1186/s12889-024-19047-y (PMC11238482; doi:10.1186/s12889-024-19047-y)
Supplement: Supplementary file 1 — Supplementary Material 1. [file 12889_2024_19047_MOESM1_ESM.docx]

**Supplementary material**

**Table and figures’ legends**

**Table S1** Prevalence of NAFLD between 1990 and 2019 in the 15- to 64-year-old age group at the global and Western Pacific Region.

**Table S2** DALYs for NAFLD between 1990 and 2019 in the 15- to 64-year-old age group at the global and Western Pacific Region.

**Table S3** Deaths from NAFLD between 1990 and 2019 in the 15- to 64-year-old age group at the global and Western Pacific Region.

**Table S4.** Annual percentage change in NAFLD incidence in each age group (Local drift) by sex from 1990 to 2019 in working-age individuals.

**Table S5.** Fitted longitudinal age effects of NAFLD incidence (per 100,000 person-years) and the corresponding 95% CIs in working-age individuals.

**Table S6.** Annual percentage change in NAFLD prevalence in each age group (Local drift) by sex from 1990 to 2019 in working-age individuals.

**Table S7.** Fitted longitudinal age effects of NAFLD prevalence (per 100 000 person-years) and the corresponding 95% CIs in working-age individuals.

**Table S8.** Annual percentage change for NAFLD DALY rate in each age group (Local drift) by sex from 1990 to 2019 in working-age individuals.

**Table S9.** Fitted longitudinal age effects of NAFLD DALY rate (per 100 000 person-years) and the corresponding 95% CIs in working-age individuals.

**Table S10.** Relative risk for NAFLD incidence rate for each period compared with the reference period (2000–2004), and the corresponding 95% CIs by sex in working-age individuals.

**Table S11.** Relative risk for NAFLD prevalence rate for each period compared with the reference period (2000–2004), and the corresponding 95% CIs by sex in working-age individuals.

**Table S12.** Relative risk for NAFLD DALY rate for each period compared with the reference period (2000–2004), and the corresponding 95% CIs by sex in working-age individuals.

**Table S13.** Relative risk for NAFLD incidence rate for each birth cohort compared with the reference cohort (1980-1989), and the corresponding 95% CIs by sex in working-age individuals.

**Table S14.** Relative risk for NAFLD prevalence rate for each birth cohort compared with the reference cohort (1980-1989), and the corresponding 95% CIs by sex in working-age individuals.

**Table S15.** Relative risk for NAFLD DALY rate for each birth cohort compared with the reference cohort (1980-1989), and the corresponding 95% CIs by sex in working-age individuals.

**Figure S1**. Trends in NAFLD prevalence, incidence, Disability-Adjusted Life-Years (DALYs), and deaths from 1990 to 2019. (A) Trends in prevalence; (B) Trends in incidence; (C) Trends in DALYs; (D) Trends in deaths.

**Figure S2**. Trends in NAFLD prevalence, incidence, Disability-Adjusted Life-Years (DALYs) and deaths from 1990 to 2019. (A) Trends in prevalence rate; (B) Trends in incidence rate; (C) Trends in DALYs rate; (D) Trends in deaths rate.

**Figure S3**. Ratio of Male to Female Prevalence, Incidence, Disability-Adjusted Life-Years (DALY), and Deaths from NAFLD in Different Age Subgroups. (A) Male-to-female Ratio of Prevalence; (B) Male-to-female Ratio of Incidence; (C) Male-to-female Ratio of DALY; (D) Male-to-female Ratio of Deaths.

**Figure S4.** Annual rates of NAFLD loss by age and gender in different Socio-demographic Index (SDI) regions of the Western Pacific region, 1990 and 2019. (A) Age-standardized Incidence Rate; (B) Age-standardized Prevalence Rate; (C) Age-standardized Disability-Adjusted Life-Years Rate; (D) Age-standardized Deaths Rate.

**Figure S5.** Trends of Age-standardized Incidence Rate (ASIR), Prevalence Rate (ASPR), and Disability-Adjusted Life-Years (DALYs) of NAFLD in different Socio-demographic Index (SDI) regions from 1990 to 2019.

**Figure S6.** Association between Age-standardized NAFLD Incidence Rate and Socio-demographic Index (SDI).

**Figure S7.** Association between Age-standardized NAFLD Disability-Adjusted Life-Years (DALYs) Rate and Socio-demographic Index (SDI).

**Figure S8.** Association between Age-standardized NAFLD Deaths Rate and Socio-demographic Index (SDI).

**Figure S9.** Temporal changes in the age distribution of NAFLD incidence among working-age individuals from 1990 to 2019.

**Figure S10.** Temporal changes in the age distribution of NAFLD Disability-Adjusted Life Years (DALYs) among working-age individuals from 1990 to 2019.

**Figure S11.** Local drift in NAFLD prevalence in the Western Pacific from 1990 to 2019.

**Figure S12.** Local drift in NAFLD Disability-Adjusted Life Years (DALYs) in the Western Pacific from 1990 to 2019.

**Figure S13.** Age effect, period effect, and cohort effect on NAFLD prevalence in the Western Pacific from 1990 to 2019.

**Figure S14.** Age effect, period effect, and cohort effect on NAFLD Disability-Adjusted Life Years (DALYs) in the Western Pacific from 1990 to 2019.

**Table S1.** Prevalence of NAFLD between 1990 and 2019 in the 15- to 64-year-old age group at the global and Western Pacific Region.

| Location | 1990 | |  | 2019 | | EAPC_95%CI |
| --- | --- | --- | --- | --- | --- | --- |
|  | Number_95%UI | ASPR |  | Number_95%UI | ASPR |  |
| Global | 473539547.2 (341732211.2-629831632.1) | 14477.6 (10447.8-19255.9) |  | 1002886383.3 (742743852.7-1298691912) | 19837.6 (14691.9-25688.8) | 1.1 (1-1.2) |
| Western Pacific Region | 141926763.8 (100909536.3-190924133.4) | 13709.2 (9747.2-18442) |  | 283234498.8 (208499833.1-367325048.3) | 20921.7 (15401.3-27133.3) | 1.48 (1.21-1.74) |
| Australia | 1047224.8 (746467.2-1404468.6) | 9336.4 (6655.1-12521.4) |  | 2103315.8 (1537202-2728611.7) | 13178.7 (9631.6-17096.6) | 1.21 (1.1-1.32) |
| Brunei Darussalam | 13313.7 (9433.9-18056.7) | 8244.8 (5842.1-11182) |  | 34174.4 (24440.9-45395.1) | 10578.7 (7565.7-14052) | 0.88 (0.86-0.91) |
| Cambodia | 888977.1 (631950.8-1197153.3) | 16811.6 (11951-22639.6) |  | 2189393.5 (1582744.5-2893996.4) | 20494.9 (14816-27090.6) | 0.64 (0.54-0.73) |
| China | 113848088.5 (80579318.3-153634793.7) | 14317.9 (10133.9-19321.6) |  | 229014015.4 (168524805.9-297137609.7) | 22469.5 (16534.6-29153.3) | 1.54 (1.21-1.88) |
| Cook Islands | 2403 (1733.7-3188.9) | 21220.7 (15310.3-28160.7) |  | 3482.3 (2630-4433.8) | 30211.2 (22816.5-38465.4) | 1.13 (1-1.26) |
| Fiji | 92211.1 (66426-122531.2) | 20319.6 (14637.6-27000.9) |  | 161063.3 (118948.4-208758.6) | 27194.8 (20083.9-35247.9) | 0.93 (0.87-1) |
| Japan | 8058598.7 (5776571.8-10742594.1) | 9181.5 (6581.5-12239.5) |  | 8175349.9 (5937937.9-10743973.7) | 10780.8 (7830.3-14168) | 0.55 (0.5-0.6) |
| Kiribati | 7960.9 (5713-10618.3) | 18838.3 (13519.1-25126.6) |  | 16281.3 (11855.5-21407) | 22424.8 (16328.9-29484.5) | 0.41 (0.24-0.58) |
| Lao People's Democratic Republic | 287792.6 (204971.9-388409.9) | 13256 (9441.2-17890.5) |  | 711789.7 (517417-940935.2) | 15466.1 (11242.7-20445) | 0.53 (0.45-0.61) |
| Malaysia | 2244564.2 (1615335.4-2984548.5) | 21552.4 (15510.5-28657.8) |  | 6035233.9 (4462469.8-7815539.3) | 28120.6 (20792.5-36415.8) | 0.89 (0.87-0.91) |
| Marshall Islands | 3514.3 (2483.7-4728.3) | 15753.6 (11133.8-21195.7) |  | 7585 (5467.2-9980.3) | 20946 (15097.6-27560.6) | 1.03 (1-1.05) |
| Micronesia (Federated States of) | 9809.7 (7034.5-13120.4) | 18301.3 (13123.7-24477.8) |  | 15315.1 (11202.6-20106.1) | 23053.8 (16863.3-30265.8) | 0.77 (0.65-0.89) |
| Mongolia | 146157.7 (103216.5-197479.8) | 12473.5 (8808.8-16853.5) |  | 361290.8 (261934.6-477391.9) | 16134.3 (11697.3-21319.1) | 0.81 (0.67-0.96) |
| Nauru | 1098.6 (786.3-1464.5) | 19535.8 (13981-26042.2) |  | 1416.3 (1025.3-1868.3) | 21900 (15853.9-28889.5) | 0.22 (0.14-0.29) |
| New Zealand | 201017 (143380.2-268683.6) | 8963 (6393.1-11980.1) |  | 355390.1 (261272.6-458699) | 12454.3 (9156-16074.6) | 1.12 (1.03-1.21) |
| Niue | 277.7 (199.6-365.3) | 21448.7 (15419.8-28217.1) |  | 306.8 (229.9-393.4) | 28331.7 (21235.1-36330.4) | 0.92 (0.81-1.03) |
| Palau | 2022.9 (1450.5-2690.2) | 20473.1 (14680.3-27227.6) |  | 3980.1 (2990.4-5066.1) | 30251.3 (22729.5-38505.9) | 1.18 (1.04-1.33) |
| Papua New Guinea | 369637.8 (262357.3-497475.4) | 16173.2 (11479.2-21766.7) |  | 1079703.3 (770470.1-1432759.6) | 18305.3 (13062.6-24291) | 0.38 (0.36-0.41) |
| Philippines | 4845000.6 (3477890.8-6463604.5) | 13563.5 (9736.3-18094.7) |  | 12037970.5 (8822853-15708520.4) | 16988.9 (12451.5-22169) | 0.78 (0.75-0.8) |
| Republic of Korea | 2578854.1 (1827300.7-3470331.8) | 8393.6 (5947.4-11295.1) |  | 4416727.4 (3185961.9-5837801) | 11398.4 (8222.1-15065.8) | 1.61 (1.22-2.01) |
| Samoa | 17985.4 (12971.9-23949) | 19796 (14277.7-26359.9) |  | 29374.3 (21585-38301.7) | 23136.9 (17001.6-30168.7) | 0.42 (0.25-0.58) |
| Singapore | 203841.7 (143325.7-276671) | 9145.9 (6430.7-12413.6) |  | 531823.3 (382310.8-707339.5) | 12639.9 (9086.4-16811.4) | 1.12 (1.09-1.15) |
| Solomon Islands | 28395.9 (20228.8-38006.2) | 16256.1 (11580.6-21757.7) |  | 74291.1 (53212.1-98271.5) | 19618.7 (14052.2-25951.4) | 0.64 (0.53-0.75) |
| Tonga | 10421.7 (7537.5-13819.5) | 19772.4 (14300.3-26218.7) |  | 14485.6 (10660.1-18928.8) | 24209.2 (17815.8-31634.9) | 0.51 (0.33-0.69) |
| Tuvalu | 1032.3 (738.1-1375.3) | 18798.2 (13440.4-25044.5) |  | 1661 (1210.1-2177.1) | 22040.9 (16058.2-28890.3) | 0.44 (0.33-0.56) |
| Vanuatu | 14476.6 (10318.2-19380.9) | 18160.4 (12943.9-24312.6) |  | 36571.7 (26584.1-48092.3) | 21435.2 (15581.3-28187.6) | 0.6 (0.59-0.61) |
| Viet Nam | 5535087.5 (3931537.3-7462239.1) | 14410 (10235.3-19427.1) |  | 13213924.7 (9595829.8-17461770.9) | 19350.9 (14052.4-25571.5) | 1.05 (0.94-1.16) |

Abbreviations: NAFLD Non-alcoholic fatty liver disease; ASPR:Age-Standardized Prevalence Rate; EAPC Estimated annual percentage change; UI Uncertainty interval.

**Table S2.** DALYs for NAFLD between 1990 and 2019 in the 15- to 64-year-old age group at the global and Western Pacific Region.

| Location | 1990 | |  | 2019 | | EAPC_95%CI |
| --- | --- | --- | --- | --- | --- | --- |
|  | Number_95%UI | ASDR |  | Number_95%UI | ASDR |  |
| Global | 1950660.7 (1135718.1-3123269.1) | 59.6 (34.7-95.5) |  | 2913514.9 (1677499.1-4646106.5) | 57.6 (33.2-91.9) | -0.34 (-0.5--0.19) |
| Western Pacific Region | 556240.9 (360554.7-826816.5) | 53.7 (34.8-79.9) |  | 515020.7 (326259.1-772241.9) | 38 (24.1-57) | -1.92 (-2.32--1.51) |
| Australia | 3827.8 (2078.6-6342.3) | 34.1 (18.5-56.5) |  | 6838.2 (4006.2-10813.7) | 42.8 (25.1-67.8) | 1.52 (1.22-1.83) |
| Brunei Darussalam | 24.8 (13.3-43.1) | 15.4 (8.3-26.7) |  | 72 (40.3-118.4) | 22.3 (12.5-36.7) | 1.02 (0.9-1.14) |
| Cambodia | 10192.7 (4900.5-18680.6) | 192.8 (92.7-353.3) |  | 19214.4 (9179.9-35343) | 179.9 (85.9-330.8) | -0.44 (-0.68--0.19) |
| China | 448133.3 (294091-662045.7) | 56.4 (37-83.3) |  | 352814.6 (227778.3-521946.6) | 34.6 (22.3-51.2) | -2.55 (-3.03--2.08) |
| Cook Islands | 5.8 (3.2-9.8) | 51.2 (28.2-86.7) |  | 7.6 (4-12.8) | 66.1 (34.9-111) | 1.05 (0.9-1.19) |
| Fiji | 173.1 (90.5-304.6) | 38.1 (19.9-67.1) |  | 288.9 (149.6-504.1) | 48.8 (25.3-85.1) | 1.06 (0.96-1.17) |
| Japan | 25610.1 (17330.6-37382.5) | 29.2 (19.7-42.6) |  | 11117.8 (7441.5-16230.6) | 14.7 (9.8-21.4) | -2.9 (-3.12--2.67) |
| Kiribati | 58.3 (23.4-120.1) | 138 (55.4-284.3) |  | 89.2 (35.2-184) | 122.8 (48.5-253.4) | -0.52 (-0.6--0.45) |
| Lao People's Democratic Republic | 1870.3 (832.9-3562.4) | 86.1 (38.4-164.1) |  | 2973.1 (1360.2-5638.5) | 64.6 (29.6-122.5) | -1.31 (-1.6--1.01) |
| Malaysia | 2858.6 (1567.2-4782.1) | 27.4 (15-45.9) |  | 8693.6 (4569.3-15029) | 40.5 (21.3-70) | 1.26 (1.06-1.47) |
| Marshall Islands | 17.6 (8-33.9) | 78.7 (36-152.1) |  | 33.6 (14.4-68) | 92.9 (39.6-187.9) | 0.57 (0.5-0.64) |
| Micronesia (Federated States of) | 49.3 (20.6-99.4) | 92 (38.5-185.5) |  | 64.8 (22.4-139.9) | 97.6 (33.7-210.6) | 0.35 (0.27-0.43) |
| Mongolia | 1630.7 (888.2-2783.8) | 139.2 (75.8-237.6) |  | 5394.6 (2963.7-8949.3) | 240.9 (132.4-399.7) | 2.01 (1.89-2.14) |
| Nauru | 5.2 (1.9-11) | 92.2 (34.2-195.3) |  | 5.8 (2.2-12.3) | 89.7 (34.6-191) | -0.11 (-0.18--0.04) |
| New Zealand | 550 (327.6-862.5) | 24.5 (14.6-38.5) |  | 900.4 (589-1315.7) | 31.6 (20.6-46.1) | 0.89 (0.84-0.95) |
| Niue | 1 (0.5-1.9) | 79.5 (38.4-147.5) |  | 0.9 (0.5-1.7) | 86.8 (42.4-156.1) | 0.16 (-0.04-0.35) |
| Palau | 5.9 (2.5-12.2) | 60.1 (24.9-123.4) |  | 12.6 (5.8-23.9) | 95.9 (44.4-181.7) | 1.87 (1.69-2.04) |
| Papua New Guinea | 594.4 (260.1-1167.9) | 26 (11.4-51.1) |  | 1663.7 (711.5-3346.1) | 28.2 (12.1-56.7) | 0.42 (0.31-0.52) |
| Philippines | 18861.6 (10638.4-31108.5) | 52.8 (29.8-87.1) |  | 38164.2 (21810.3-62306.9) | 53.9 (30.8-87.9) | -0.15 (-0.33-0.03) |
| Republic of Korea | 10256.1 (6290.2-15877.5) | 33.4 (20.5-51.7) |  | 12189.1 (7383.3-19017.6) | 31.5 (19.1-49.1) | -0.68 (-1.2--0.17) |
| Samoa | 52 (24.3-99.3) | 57.3 (26.7-109.3) |  | 71.8 (32.9-135.6) | 56.6 (25.9-106.8) | 0.07 (0.01-0.12) |
| Singapore | 233.8 (139.8-369.3) | 10.5 (6.3-16.6) |  | 379 (229.6-579.4) | 9 (5.5-13.8) | -0.76 (-0.92--0.61) |
| Solomon Islands | 188.7 (86.7-359.3) | 108 (49.6-205.7) |  | 438.9 (209-805.8) | 115.9 (55.2-212.8) | 0.43 (0.32-0.54) |
| Tonga | 51.7 (27.7-86.8) | 98.1 (52.6-164.6) |  | 71.3 (37.7-121.7) | 119.2 (63-203.3) | 0.57 (0.35-0.78) |
| Tuvalu | 5.1 (2.2-10.2) | 93.4 (40.7-185.1) |  | 6 (2.8-11.4) | 79.4 (37-151.1) | -0.42 (-0.52--0.33) |
| Vanuatu | 58.6 (24.9-118.2) | 73.6 (31.2-148.2) |  | 136.4 (59.5-268.4) | 79.9 (34.9-157.3) | 0.08 (-0.06-0.22) |
| Viet Nam | 23248.5 (10453.5-44953.7) | 60.5 (27.2-117) |  | 41954.2 (18865.5-81062.6) | 61.4 (27.6-118.7) | -0.15 (-0.79-0.5) |

Abbreviations: NAFLD Non-alcoholic fatty liver disease; DALYs:Disability-Adjusted Life Years; ASDR: age-standardized DALY rate; EAPC Estimated annual percentage change; UI Uncertainty interval.

**Table S3.** Deaths from NAFLD between 1990 and 2019 in the 15- to 64-year-old age group at the global and Western Pacific Region.

| Location | 1990 | |  | 2019 | | EAPC_95%CI |
| --- | --- | --- | --- | --- | --- | --- |
|  | Number_95%UI | ASR |  | Number_95%UI | ASR |  |
| Global | 49236.1 (28421.8-79030.2) | 1.5 (0.9-2.4) |  | 75325 (43115.8-120282.2) | 1.5 (0.9-2.4) | -0.23 (-0.4--0.06) |
| Western Pacific Region | 14251.6 (9224.6-21191.9) | 1.4 (0.9-2) |  | 13676 (8608.1-20518.9) | 1 (0.6-1.5) | -1.76 (-2.17--1.34) |
| Australia | 101.5 (54.7-168.7) | 0.9 (0.5-1.5) |  | 190.5 (111.4-300.6) | 1.2 (0.7-1.9) | 1.76 (1.42-2.09) |
| Brunei Darussalam | 0.6 (0.3-1.1) | 0.4 (0.2-0.7) |  | 1.9 (1.1-3.1) | 0.6 (0.3-1) | 1.33 (1.19-1.46) |
| Cambodia | 235.4 (112.1-431.8) | 4.5 (2.1-8.2) |  | 468.6 (221.5-866.6) | 4.4 (2.1-8.1) | -0.24 (-0.51-0.04) |
| China | 11449.2 (7483.7-16932.7) | 1.4 (0.9-2.1) |  | 9419.5 (6023.9-13973.2) | 0.9 (0.6-1.4) | -2.35 (-2.83--1.87) |
| Cook Islands | 0.2 (0.1-0.3) | 1.3 (0.7-2.2) |  | 0.2 (0.1-0.4) | 1.8 (1-3.1) | 1.17 (1.01-1.34) |
| Fiji | 4.1 (2.2-7.2) | 0.9 (0.5-1.6) |  | 7.4 (3.8-12.9) | 1.3 (0.6-2.2) | 1.34 (1.23-1.46) |
| Japan | 739.1 (500.5-1080.2) | 0.8 (0.6-1.2) |  | 318.4 (211.8-467.1) | 0.4 (0.3-0.6) | -2.93 (-3.16--2.69) |
| Kiribati | 1.4 (0.6-2.8) | 3.3 (1.4-6.6) |  | 2.1 (0.9-4.4) | 3 (1.2-6) | -0.5 (-0.6--0.4) |
| Lao People's Democratic Republic | 46.9 (21.3-88.5) | 2.2 (1-4.1) |  | 74.7 (34-142) | 1.6 (0.7-3.1) | -1.33 (-1.66--1) |
| Malaysia | 74.5 (40.7-124.7) | 0.7 (0.4-1.2) |  | 237.3 (123.7-411.3) | 1.1 (0.6-1.9) | 1.5 (1.28-1.71) |
| Marshall Islands | 0.4 (0.2-0.8) | 1.8 (0.9-3.5) |  | 0.8 (0.4-1.6) | 2.3 (1-4.5) | 0.79 (0.7-0.88) |
| Micronesia (Federated States of) | 1.2 (0.5-2.3) | 2.2 (0.9-4.3) |  | 1.6 (0.6-3.5) | 2.5 (0.9-5.3) | 0.63 (0.5-0.76) |
| Mongolia | 42.4 (23.4-71.4) | 3.6 (2-6.1) |  | 144.5 (79.3-238.6) | 6.5 (3.5-10.7) | 2.12 (2.01-2.24) |
| Nauru | 0.1 (0-0.3) | 2.1 (0.8-4.5) |  | 0.1 (0.1-0.3) | 2.1 (0.8-4.4) | -0.07 (-0.13-0) |
| New Zealand | 14.8 (8.7-23.4) | 0.7 (0.4-1) |  | 25.4 (16.5-37.3) | 0.9 (0.6-1.3) | 1.11 (1.05-1.17) |
| Niue | 0 (0-0) | 2 (1-3.7) |  | 0 (0-0) | 2.3 (1.2-4.1) | 0.4 (0.2-0.6) |
| Palau | 0.1 (0.1-0.3) | 1.4 (0.6-2.9) |  | 0.3 (0.2-0.6) | 2.5 (1.2-4.7) | 2.2 (2.05-2.34) |
| Papua New Guinea | 13.6 (6-26.9) | 0.6 (0.3-1.2) |  | 38.6 (16.4-77.7) | 0.7 (0.3-1.3) | 0.47 (0.35-0.59) |
| Philippines | 461.8 (256.9-769.8) | 1.3 (0.7-2.2) |  | 973.2 (551.8-1589.4) | 1.4 (0.8-2.2) | -0.01 (-0.2-0.19) |
| Republic of Korea | 263.4 (160.9-408.2) | 0.9 (0.5-1.3) |  | 349.3 (211.1-543) | 0.9 (0.5-1.4) | -0.33 (-0.87-0.21) |
| Samoa | 1.3 (0.6-2.4) | 1.4 (0.7-2.7) |  | 1.8 (0.8-3.4) | 1.4 (0.7-2.6) | 0.16 (0.09-0.23) |
| Singapore | 6.3 (3.8-10) | 0.3 (0.2-0.4) |  | 11 (6.6-16.9) | 0.3 (0.2-0.4) | -0.45 (-0.63--0.27) |
| Solomon Islands | 4.4 (2-8.3) | 2.5 (1.1-4.8) |  | 10 (4.7-18.3) | 2.6 (1.2-4.8) | 0.33 (0.24-0.42) |
| Tonga | 1.3 (0.7-2.3) | 2.6 (1.4-4.3) |  | 1.9 (1-3.2) | 3.1 (1.7-5.3) | 0.55 (0.35-0.76) |
| Tuvalu | 0.1 (0.1-0.3) | 2.3 (1-4.6) |  | 0.2 (0.1-0.3) | 2 (0.9-3.8) | -0.41 (-0.52--0.3) |
| Vanuatu | 1.4 (0.6-2.8) | 1.7 (0.7-3.5) |  | 3.3 (1.5-6.4) | 1.9 (0.9-3.8) | 0.18 (0.03-0.33) |
| Viet Nam | 591 (265.2-1143) | 1.5 (0.7-3) |  | 1094 (488.2-2124.3) | 1.6 (0.7-3.1) | -0.05 (-0.75-0.66) |

Abbreviations: NAFLD Non-alcoholic fatty liver disease; ASR:Age-Standardized Rate; EAPC Estimated annual percentage change; UI Uncertainty interval.

**Table S4.** Annual percentage change in NAFLD incidence in each age group (Local drift) by sex from 1990 to 2019 in working-age individuals.

| **Age group** | **Both** | **Male** | **Female** |
| --- | --- | --- | --- |
| 15-19 | -1.06 (-2.63, 0.54) | -0.91 (-2.61, 0.81) | -1.26 (-3.19, 0.71) |
| 20-24 | -0.56 (-1.37, 0.25) | -0.39 (-1.27, 0.49) | -0.78 (-1.77, 0.22) |
| 25-29 | -0.3 (-0.79, 0.18) | -0.12 (-0.65, 0.41) | -0.52 (-1.12, 0.08) |
| 30-34 | -0.21 (-0.56, 0.14) | -0.01 (-0.4, 0.37) | -0.45 (-0.88, -0.01) |
| 35-39 | -0.23 (-0.51, 0.06) | -0.04 (-0.35, 0.27) | -0.45 (-0.8, -0.11) |
| 40-44 | -0.29 (-0.53, -0.04) | -0.17 (-0.45, 0.11) | -0.45 (-0.73, -0.17) |
| 45-49 | -0.46 (-0.7, -0.22) | -0.54 (-0.83, -0.24) | -0.48 (-0.74, -0.23) |
| 50-54 | -0.77 (-1.03, -0.5) | -1.13 (-1.48, -0.77) | -0.61 (-0.88, -0.35) |
| 55-59 | -1.17 (-1.49, -0.85) | -1.76 (-2.2, -1.32) | -0.88 (-1.19, -0.56) |
| 60-64 | -1.47 (-1.93, -1) | -2.1 (-2.72, -1.47) | -1.11 (-1.57, -0.66) |

NAFLD Non-alcoholic fatty liver disease.

**Table S5.** Fitted longitudinal age effects of NAFLD incidence (per 100,000 person-years) and the corresponding 95% CIs in working-age individuals.

| **Age group** | **Both** | **Male** | **Female** |
| --- | --- | --- | --- |
| 15-19 | 0.16 (0.13, 0.19) | 0.18 (0.15, 0.22) | 0.13 (0.11, 0.16) |
| 20-24 | 0.4 (0.35, 0.44) | 0.46 (0.41, 0.52) | 0.33 (0.29, 0.38) |
| 25-29 | 0.87 (0.8, 0.94) | 1.05 (0.96, 1.15) | 0.69 (0.62, 0.76) |
| 30-34 | 1.83 (1.72, 1.95) | 2.33 (2.17, 2.5) | 1.34 (1.24, 1.45) |
| 35-39 | 3.18 (3.02, 3.36) | 3.99 (3.76, 4.24) | 2.42 (2.27, 2.58) |
| 40-44 | 4.59 (4.36, 4.83) | 5.21 (4.91, 5.52) | 4.05 (3.83, 4.29) |
| 45-49 | 5.42 (5.16, 5.69) | 5.17 (4.87, 5.49) | 5.75 (5.45, 6.06) |
| 50-54 | 5.23 (4.97, 5.5) | 4 (3.74, 4.27) | 6.48 (6.14, 6.84) |
| 55-59 | 4.43 (4.18, 4.69) | 2.96 (2.74, 3.19) | 5.92 (5.58, 6.28) |
| 60-64 | 3.63 (3.4, 3.88) | 2.64 (2.43, 2.87) | 4.63 (4.32, 4.97) |

NAFLD Non-alcoholic fatty liver disease; CI, confidence interval.

**Table S6.** Annual percentage change in NAFLD prevalence in each age group (Local drift) by sex from 1990 to 2019 in working-age individuals.

| **Age group** | **Both** | **Male** | **Female** |
| --- | --- | --- | --- |
| 15-19 | 0.94 (0.77, 1.1) | 1 (0.83, 1.18) | 0.68 (0.42, 0.94) |
| 20-24 | 1.1 (1, 1.2) | 1.24 (1.13, 1.35) | 0.84 (0.7, 0.98) |
| 25-29 | 1.08 (1.01, 1.16) | 1.27 (1.19, 1.36) | 0.84 (0.75, 0.94) |
| 30-34 | 1.01 (0.94, 1.07) | 1.19 (1.11, 1.26) | 0.8 (0.71, 0.88) |
| 35-39 | 0.89 (0.83, 0.95) | 1.06 (0.99, 1.13) | 0.7 (0.62, 0.78) |
| 40-44 | 0.85 (0.79, 0.91) | 0.98 (0.91, 1.05) | 0.7 (0.63, 0.78) |
| 45-49 | 0.85 (0.79, 0.91) | 0.92 (0.84, 0.99) | 0.79 (0.71, 0.87) |
| 50-54 | 0.84 (0.77, 0.9) | 0.83 (0.75, 0.91) | 0.87 (0.79, 0.96) |
| 55-59 | 0.78 (0.71, 0.86) | 0.75 (0.66, 0.84) | 0.83 (0.73, 0.93) |
| 60-64 | 0.73 (0.62, 0.83) | 0.72 (0.59, 0.85) | 0.72 (0.59, 0.86) |

CI, confidence interval.

**Table S7.** Fitted longitudinal age effects of NAFLD prevalence (per 100 000 person-years) and the corresponding 95% CIs in working-age individuals.

| **Age group** | **Both** | **Male** | **Female** |
| --- | --- | --- | --- |
| 15-19 | 3731.5 (3656.11, 3808.45) | 4713.69 (4609.76, 4819.96) | 2608.44 (2528.96, 2690.42) |
| 20-24 | 7315 (7204.93, 7426.75) | 8268.64 (8125.1, 8414.71) | 6250.58 (6121.65, 6382.22) |
| 25-29 | 11518.57 (11370.54, 11668.53) | 12180.68 (11995.09, 12369.15) | 10771.81 (10588.85, 10957.94) |
| 30-34 | 14962.11 (14781.89, 15144.53) | 15726.72 (15500.94, 15955.78) | 14083.57 (13861.01, 14309.71) |
| 35-39 | 17023.06 (16823.14, 17225.34) | 17989.12 (17737.08, 18244.73) | 15927.58 (15682.72, 16176.25) |
| 40-44 | 18338.77 (18121.4, 18558.75) | 19179.95 (18907.69, 19456.13) | 17411.48 (17142.88, 17684.29) |
| 45-49 | 20748.5 (20505.77, 20994.1) | 20970.87 (20673.75, 21272.27) | 20553.82 (20245.16, 20867.2) |
| 50-54 | 22674.99 (22405.61, 22947.61) | 22146.15 (21824.93, 22472.09) | 23367.22 (23013.77, 23726.1) |
| 55-59 | 23506.11 (23218.05, 23797.74) | 22551.59 (22213.46, 22894.86) | 24741.55 (24355.69, 25133.53) |
| 60-64 | 24138.99 (23830.37, 24451.6) | 22877.33 (22518.43, 23241.95) | 25770.56 (25351.42, 26196.64) |

CI, confidence interval.

**Table S8.** Annual percentage change for NAFLD DALY rate in each age group (Local drift) by sex from 1990 to 2019 in working-age individuals.

| **Age group** | **Both** | **Male** | **Female** |
| --- | --- | --- | --- |
| 15-19 | -2.95 (-3.76, -2.12) | -2.49 (-3.48, -1.48) | -3.49 (-5.29, -1.66) |
| 20-24 | -2.81 (-3.26, -2.35) | -2.14 (-2.69, -1.59) | -3.67 (-4.69, -2.64) |
| 25-29 | -2.98 (-3.28, -2.68) | -2.26 (-2.61, -1.91) | -4.09 (-4.79, -3.37) |
| 30-34 | -3.04 (-3.26, -2.81) | -2.35 (-2.61, -2.1) | -4.27 (-4.83, -3.7) |
| 35-39 | -3.16 (-3.34, -2.99) | -2.52 (-2.71, -2.32) | -4.49 (-4.95, -4.02) |
| 40-44 | -3.3 (-3.44, -3.16) | -2.62 (-2.77, -2.46) | -4.7 (-5.07, -4.32) |
| 45-49 | -3.28 (-3.41, -3.16) | -2.62 (-2.75, -2.48) | -4.59 (-4.91, -4.27) |
| 50-54 | -3.23 (-3.34, -3.11) | -2.63 (-2.76, -2.5) | -4.29 (-4.58, -4) |
| 55-59 | -3.18 (-3.3, -3.06) | -2.63 (-2.77, -2.49) | -4.04 (-4.34, -3.75) |
| 60-64 | -3.03 (-3.19, -2.87) | -2.52 (-2.71, -2.33) | -3.7 (-4.07, -3.34) |

DALY, Disability-Adjusted Life Years; CI, confidence interval.

**Table S9.** Fitted longitudinal age effects of NAFLD DALY rate (per 100 000 person-years) and the corresponding 95% CIs in working-age individuals.

| **Age group** | **Both** | **Male** | **Female** |
| --- | --- | --- | --- |
| 15-19 | 2.89 (2.66, 3.13) | 2.89 (2.6, 3.2) | 2.89 (2.42, 3.45) |
| 20-24 | 5.9 (5.56, 6.26) | 5.93 (5.51, 6.38) | 5.92 (5.2, 6.74) |
| 25-29 | 10.5 (10.02, 11) | 11.94 (11.31, 12.61) | 9.1 (8.16, 10.16) |
| 30-34 | 19.87 (19.14, 20.62) | 24.3 (23.29, 25.35) | 15.38 (14.03, 16.86) |
| 35-39 | 32.74 (31.73, 33.78) | 43.89 (42.4, 45.43) | 20.89 (19.25, 22.67) |
| 40-44 | 53.56 (52.12, 55.04) | 71.99 (69.86, 74.18) | 33.74 (31.44, 36.22) |
| 45-49 | 76.08 (74.2, 78) | 99.79 (97.06, 102.6) | 50.41 (47.3, 53.72) |
| 50-54 | 101.03 (98.58, 103.54) | 126.94 (123.48, 130.49) | 73.02 (68.68, 77.63) |
| 55-59 | 118.32 (115.3, 121.41) | 141.46 (137.35, 145.69) | 94.04 (88.31, 100.13) |
| 60-64 | 135.07 (131.27, 138.99) | 151.37 (146.42, 156.49) | 119.48 (111.65, 127.86) |

DALY, Disability-Adjusted Life Years; CI, confidence interval.

**Table S10.** Relative risk for NAFLD incidence rate for each period compared with the reference period (2000–2004), and the corresponding 95% CIs by sex in working-age individuals.

| **Periods** | **Both** | **Male** | **Female** |
| --- | --- | --- | --- |
| 1990-1994 | 1.13 (1.07, 1.2) | 1.09 (1.03, 1.17) | 1.18 (1.11, 1.25) |
| 1995-1999 | 1.09 (1.04, 1.15) | 1.08 (1.02, 1.15) | 1.11 (1.05, 1.18) |
| 2000-2004 | 1 (1, 1) | 1 (1, 1) | 1 (1, 1) |
| 2005-2009 | 0.91 (0.87, 0.96) | 0.91 (0.85, 0.96) | 0.91 (0.87, 0.97) |
| 2010-2014 | 0.95 (0.91, 1) | 0.93 (0.87, 0.98) | 0.97 (0.91, 1.02) |
| 2015-2019 | 1.02 (0.97, 1.08) | 0.99 (0.93, 1.05) | 1.04 (0.98, 1.1) |

CI, confidence interval.

**Table S11.** Relative risk for NAFLD prevalence rate for each period compared with the reference period (2000–2004), and the corresponding 95% CIs by sex in working-age individuals.

| **Periods** | **Both** | **Male** | **Female** |
| --- | --- | --- | --- |
| 1990-1994 | 1 (0.99, 1.02) | 1.06 (1.04, 1.07) | 0.94 (0.93, 0.96) |
| 1995-1999 | 1.03 (1.02, 1.04) | 1.06 (1.04, 1.07) | 1 (0.98, 1.01) |
| 2000-2004 | 1 (1, 1) | 1 (1, 1) | 1 (1, 1) |
| 2005-2009 | 1.01 (1, 1.02) | 1.04 (1.03, 1.06) | 0.98 (0.96, 0.99) |
| 2010-2014 | 1.16 (1.15, 1.17) | 1.23 (1.22, 1.25) | 1.08 (1.06, 1.09) |
| 2015-2019 | 1.28 (1.26, 1.29) | 1.36 (1.34, 1.37) | 1.18 (1.17, 1.2) |

CI, confidence interval.

**Table S12.** Relative risk for NAFLD DALY rate for each period compared with the reference period (2000–2004), and the corresponding 95% CIs by sex in working-age individuals.

| **Periods** | **Both** | **Male** | **Female** |
| --- | --- | --- | --- |
| 1990-1994 | 1.39 (1.35, 1.43) | 1.31 (1.27, 1.36) | 1.52 (1.42, 1.63) |
| 1995-1999 | 1.28 (1.24, 1.31) | 1.22 (1.19, 1.26) | 1.37 (1.29, 1.46) |
| 2000-2004 | 1 (1, 1) | 1 (1, 1) | 1 (1, 1) |
| 2005-2009 | 0.76 (0.74, 0.78) | 0.8 (0.78, 0.83) | 0.7 (0.65, 0.74) |
| 2010-2014 | 0.68 (0.66, 0.7) | 0.74 (0.71, 0.76) | 0.59 (0.55, 0.64) |
| 2015-2019 | 0.7 (0.68, 0.72) | 0.77 (0.75, 0.79) | 0.6 (0.56, 0.64) |

DALY, Disability-Adjusted Life Years; CI, confidence interval.

**Table S13.** Relative risk for NAFLD incidence rate for each birth cohort compared with the reference cohort (1980-1989), and the corresponding 95% CIs by sex in working-age individuals.

| **Cohorts** | **Both** | **Male** | **Female** |
| --- | --- | --- | --- |
| 1925-1934 | 1.52 (1.28, 1.81) | 1.66 (1.34, 2.07) | 1.5 (1.24, 1.81) |
| 1930-1939 | 1.41 (1.23, 1.62) | 1.55 (1.31, 1.83) | 1.39 (1.19, 1.62) |
| 1935-1944 | 1.28 (1.13, 1.44) | 1.35 (1.17, 1.56) | 1.28 (1.11, 1.48) |
| 1940-1949 | 1.17 (1.04, 1.32) | 1.17 (1.03, 1.34) | 1.21 (1.05, 1.4) |
| 1945-1954 | 1.1 (0.99, 1.23) | 1.06 (0.94, 1.2) | 1.17 (1.02, 1.34) |
| 1950-1959 | 1.07 (0.96, 1.19) | 1.02 (0.91, 1.15) | 1.14 (0.99, 1.3) |
| 1955-1964 | 1.05 (0.94, 1.17) | 1 (0.89, 1.13) | 1.11 (0.97, 1.26) |
| 1960-1969 | 1.05 (0.94, 1.16) | 1.01 (0.9, 1.13) | 1.1 (0.96, 1.25) |
| 1965-1974 | 1.03 (0.93, 1.15) | 1.01 (0.9, 1.12) | 1.07 (0.94, 1.21) |
| 1970-1979 | 1.02 (0.92, 1.13) | 1.01 (0.9, 1.13) | 1.04 (0.91, 1.18) |
| 1975-1984 | 1.01 (0.9, 1.13) | 1 (0.89, 1.13) | 1.01 (0.88, 1.17) |
| 1980-1989 | 1 (1, 1) | 1 (1, 1) | 1 (1, 1) |
| 1985-1994 | 0.96 (0.82, 1.13) | 0.97 (0.82, 1.15) | 0.95 (0.78, 1.16) |
| 1990-1999 | 0.88 (0.68, 1.15) | 0.9 (0.68, 1.19) | 0.86 (0.62, 1.19) |
| 1995-2004 | 0.77 (0.45, 1.3) | 0.79 (0.45, 1.39) | 0.74 (0.39, 1.43) |

CI, confidence interval.

**Table S14.** Relative risk for NAFLD prevalence rate for each birth cohort compared with the reference cohort (1980-1989), and the corresponding 95% CIs by sex in working-age individuals.

| **Cohorts** | **Both** | **Male** | **Female** |
| --- | --- | --- | --- |
| 1925-1934 | 0.62 (0.6, 0.65) | 0.59 (0.56, 0.62) | 0.67 (0.64, 0.7) |
| 1930-1939 | 0.64 (0.62, 0.66) | 0.61 (0.59, 0.64) | 0.68 (0.65, 0.7) |
| 1935-1944 | 0.66 (0.64, 0.67) | 0.64 (0.62, 0.65) | 0.68 (0.66, 0.71) |
| 1940-1949 | 0.68 (0.67, 0.7) | 0.65 (0.64, 0.67) | 0.72 (0.7, 0.74) |
| 1945-1954 | 0.71 (0.7, 0.73) | 0.68 (0.66, 0.69) | 0.76 (0.74, 0.78) |
| 1950-1959 | 0.75 (0.74, 0.76) | 0.71 (0.69, 0.73) | 0.8 (0.78, 0.82) |
| 1955-1964 | 0.77 (0.76, 0.79) | 0.74 (0.73, 0.76) | 0.81 (0.79, 0.84) |
| 1960-1969 | 0.81 (0.8, 0.82) | 0.78 (0.76, 0.79) | 0.85 (0.83, 0.87) |
| 1965-1974 | 0.85 (0.83, 0.86) | 0.82 (0.81, 0.84) | 0.87 (0.86, 0.89) |
| 1970-1979 | 0.88 (0.87, 0.9) | 0.87 (0.85, 0.88) | 0.91 (0.88, 0.93) |
| 1975-1984 | 0.94 (0.92, 0.95) | 0.93 (0.91, 0.94) | 0.95 (0.93, 0.97) |
| 1980-1989 | 1 (1, 1) | 1 (1, 1) | 1 (1, 1) |
| 1985-1994 | 1.06 (1.03, 1.08) | 1.06 (1.04, 1.09) | 1.04 (1.01, 1.07) |
| 1990-1999 | 1.1 (1.07, 1.13) | 1.1 (1.07, 1.14) | 1.07 (1.02, 1.11) |
| 1995-2004 | 1.1 (1.04, 1.16) | 1.09 (1.03, 1.16) | 1.06 (0.98, 1.15) |

CI, confidence interval.

**Table S15.**Relative risk for NAFLD DALY rate for each birth cohort compared with the reference cohort (1980-1989), and the corresponding 95% CIs by sex in working-age individuals.

| **Cohorts** | **Both** | **Male** | **Female** |
| --- | --- | --- | --- |
| 1925-1934 | 5.55 (5.11, 6.03) | 3.82 (3.48, 4.2) | 10.33 (8.44, 12.64) |
| 1930-1939 | 4.88 (4.53, 5.26) | 3.48 (3.2, 3.79) | 8.66 (7.18, 10.44) |
| 1935-1944 | 4.17 (3.88, 4.49) | 3.11 (2.86, 3.37) | 7 (5.83, 8.4) |
| 1940-1949 | 3.56 (3.31, 3.82) | 2.68 (2.47, 2.9) | 5.9 (4.93, 7.07) |
| 1945-1954 | 3.06 (2.85, 3.29) | 2.33 (2.15, 2.52) | 5.04 (4.22, 6.01) |
| 1950-1959 | 2.59 (2.41, 2.77) | 2.05 (1.9, 2.22) | 3.95 (3.31, 4.7) |
| 1955-1964 | 2.16 (2.02, 2.32) | 1.8 (1.67, 1.95) | 2.97 (2.5, 3.54) |
| 1960-1969 | 1.85 (1.73, 1.98) | 1.59 (1.47, 1.72) | 2.39 (2.02, 2.84) |
| 1965-1974 | 1.55 (1.45, 1.66) | 1.37 (1.27, 1.48) | 1.89 (1.59, 2.24) |
| 1970-1979 | 1.33 (1.24, 1.42) | 1.2 (1.11, 1.3) | 1.52 (1.28, 1.82) |
| 1975-1984 | 1.16 (1.08, 1.26) | 1.11 (1.01, 1.2) | 1.24 (1.02, 1.5) |
| 1980-1989 | 1 (1, 1) | 1 (1, 1) | 1 (1, 1) |
| 1985-1994 | 0.86 (0.77, 0.95) | 0.88 (0.78, 0.99) | 0.85 (0.66, 1.09) |
| 1990-1999 | 0.77 (0.66, 0.9) | 0.79 (0.66, 0.95) | 0.76 (0.53, 1.07) |
| 1995-2004 | 0.62 (0.47, 0.81) | 0.63 (0.45, 0.88) | 0.61 (0.33, 1.13) |

DALY, Disability-Adjusted Life Years; CI, confidence interval.

**Figure S1**. Trends in NAFLD prevalence, incidence, Disability-Adjusted Life-Years (DALYs), and deaths from 1990 to 2019. (A) Trends in prevalence; (B) Trends in incidence; (C) Trends in DALYs; (D) Trends in deaths.

**Figure S2**. Trends in NAFLD prevalence, incidence, Disability-Adjusted Life-Years (DALYs) and deaths from 1990 to 2019. (A) Trends in prevalence rate; (B) Trends in incidence rate; (C) Trends in DALYs rate; (D) Trends in deaths rate.

**Figure S3**. Ratio of Male to Female Prevalence, Incidence, Disability-Adjusted Life-Years (DALY), and Deaths from NAFLD in Different Age Subgroups. (A) Male-to-female Ratio of Prevalence; (B) Male-to-female Ratio of Incidence; (C) Male-to-female Ratio of DALY; (D) Male-to-female Ratio of Deaths.

**Figure S4.** Annual rates of NAFLD loss by age and gender in different Socio-demographic Index (SDI) regions of the Western Pacific region, 1990 and 2019. (A) Age-standardized Incidence Rate; (B) Age-standardized Prevalence Rate; (C) Age-standardized Disability-Adjusted Life-Years Rate; (D) Age-standardized Deaths Rate.

**Figure S5.** Trends of Age-standardized Incidence Rate (ASIR), Prevalence Rate (ASPR), and Disability-Adjusted Life-Years (DALYs) of NAFLD in different Socio-demographic Index (SDI) regions from 1990 to 2019.

**Figure S6.** Association between Age-standardized NAFLD Incidence Rate and Socio-demographic Index (SDI).

**Figure S7.** Association between Age-standardized NAFLD Disability-Adjusted Life-Years (DALYs) Rate and Socio-demographic Index (SDI).

**Figure S8.** Association between Age-standardized NAFLD Deaths Rate and Socio-demographic Index (SDI).


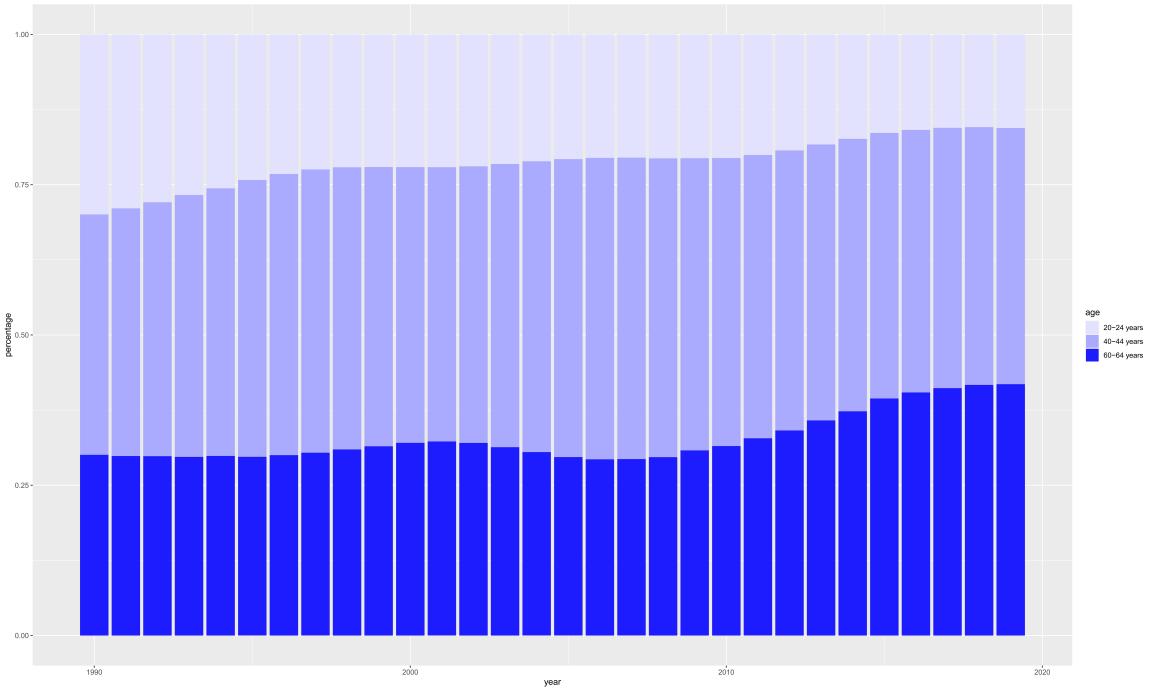


**Figure S9.** Temporal changes in the age distribution of NAFLD incidence among working-age individuals from 1990 to 2019.


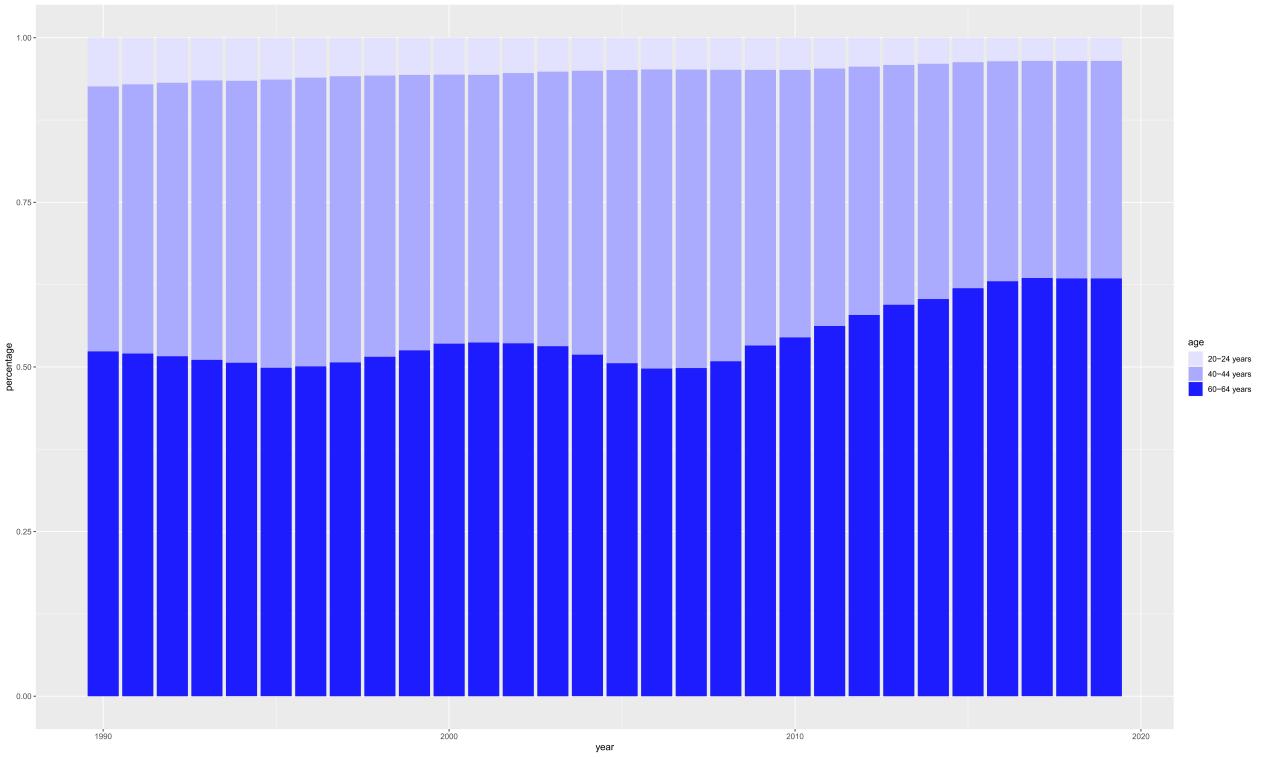


**Figure S10.** Temporal changes in the age distribution of NAFLD Disability-Adjusted Life Years (DALYs) among working-age individuals from 1990 to 2019.


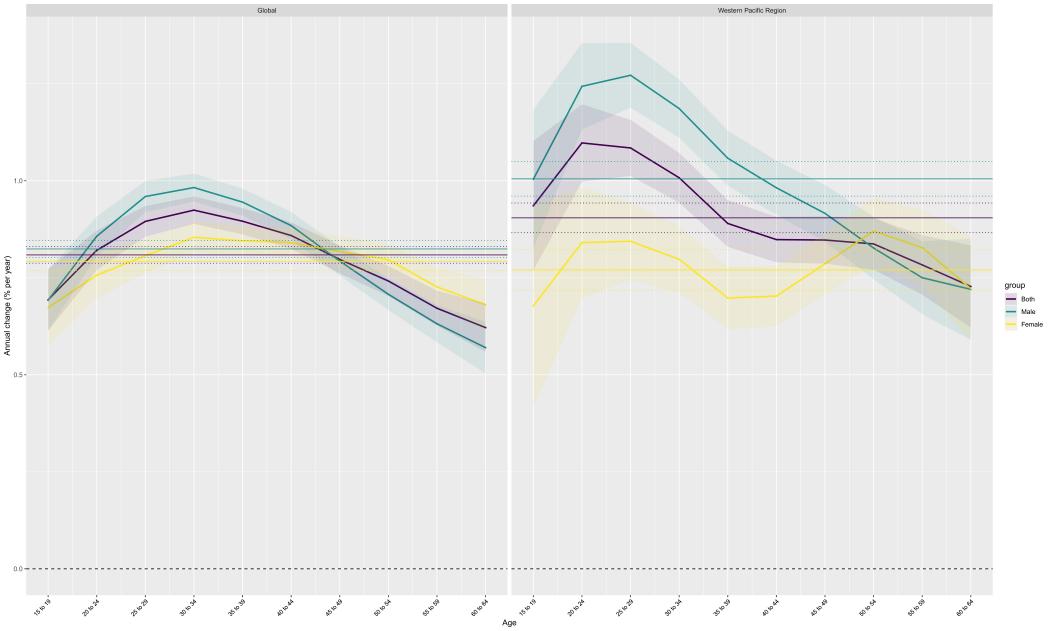


**Figure S11.** Local drift in NAFLD prevalence in the Western Pacific from 1990 to 2019.


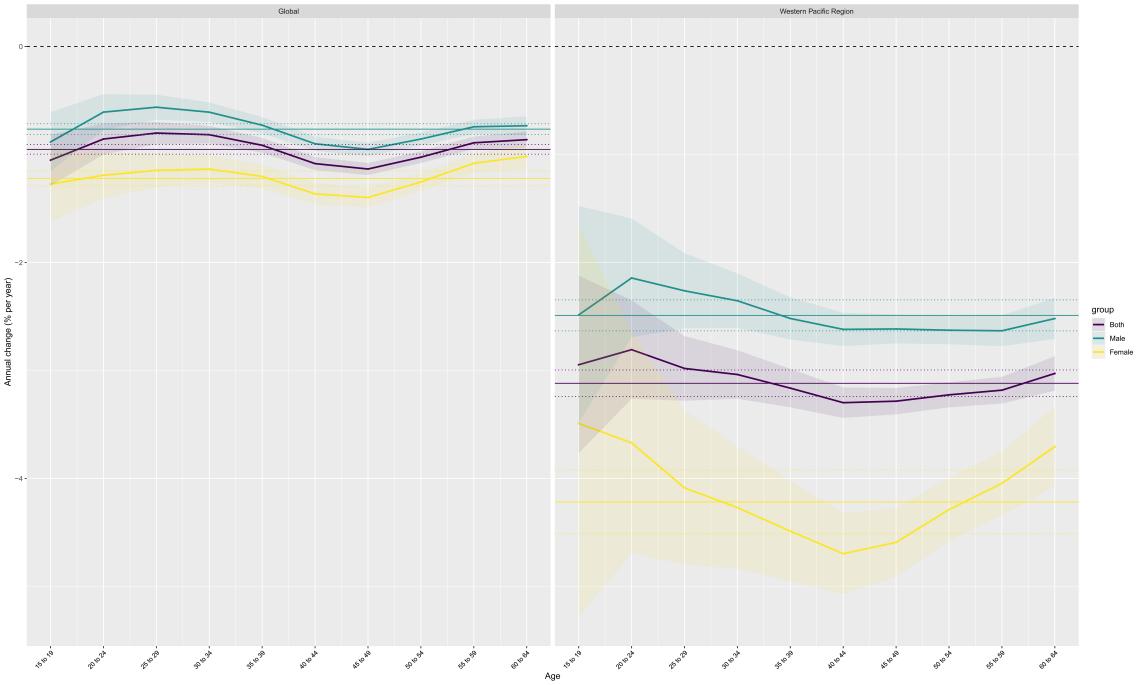


**Figure S12.** Local drift in NAFLD Disability-Adjusted Life Years (DALYs) in the Western Pacific from 1990 to 2019.

**
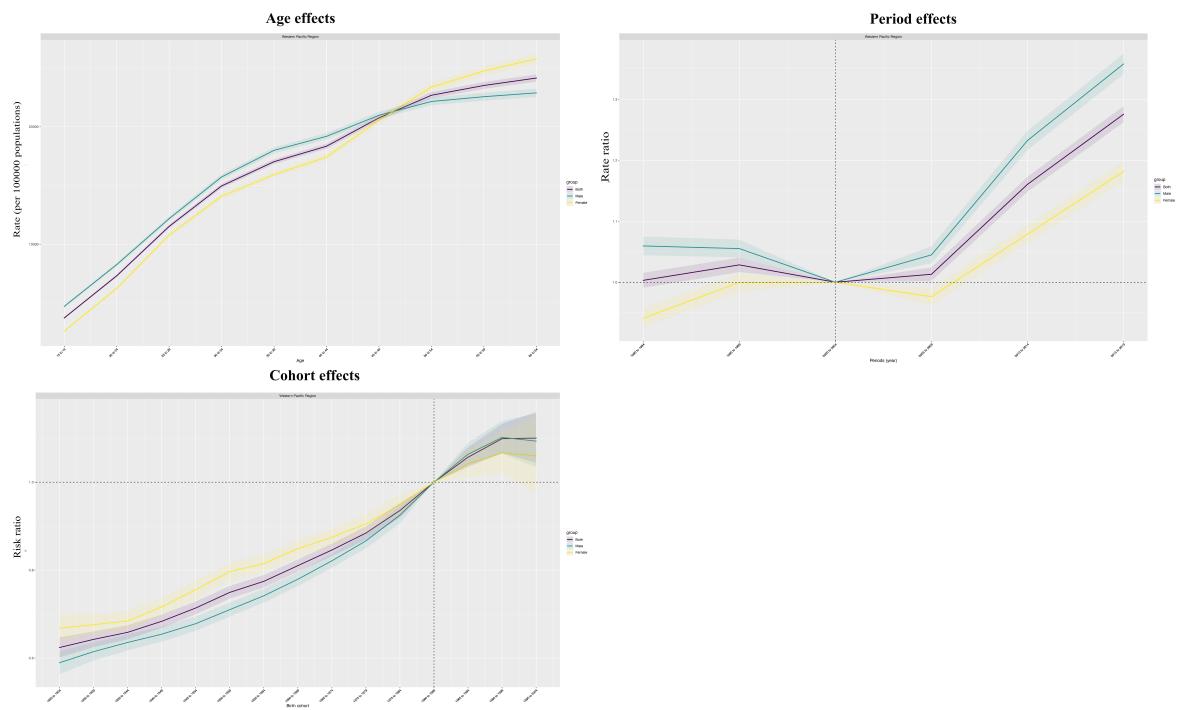
**

**Figure S13.** Age effect, period effect, and cohort effect on NAFLD prevalence in the Western Pacific from 1990 to 2019.


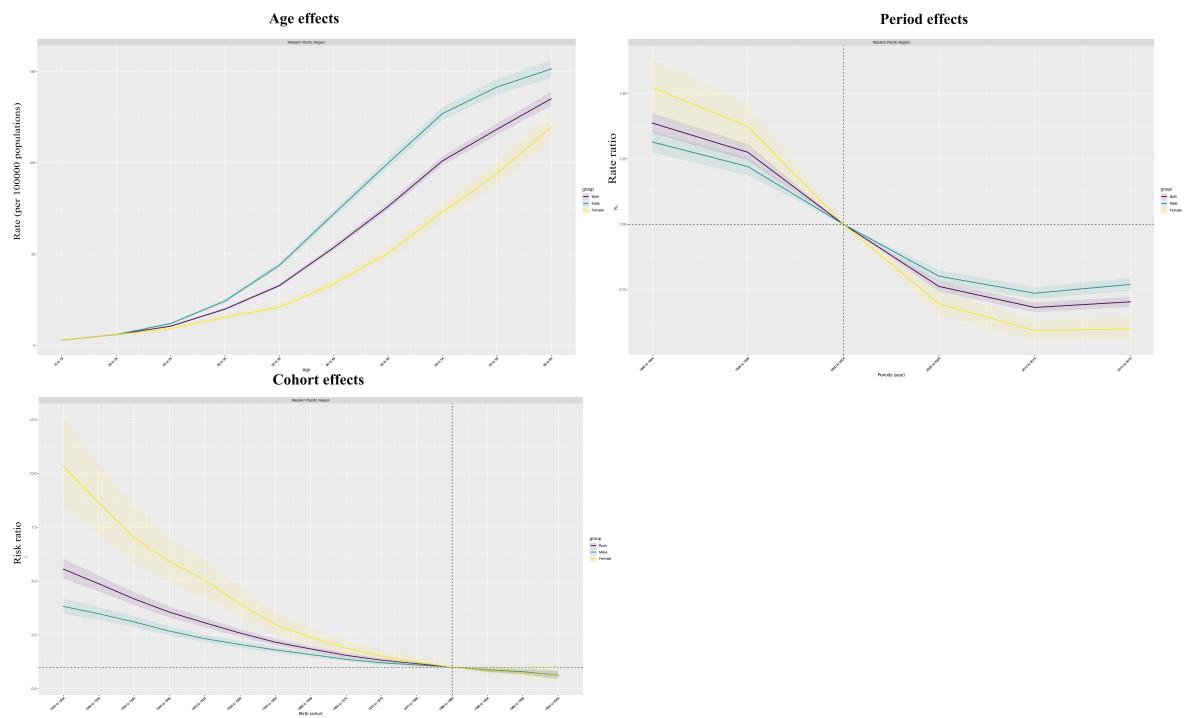


**Figure S14.** Age effect, period effect, and cohort effect on NAFLD Disability-Adjusted Life Years (DALYs) in the Western Pacific from 1990 to 2019.
